# Stable Gastric Pentadecapeptide BPC 157 and Wound Healing

**DOI:** 10.3389/fphar.2021.627533

**Published:** 2021-06-29

**Authors:** Sven Seiwerth, Marija Milavic, Jaksa Vukojevic, Slaven Gojkovic, Ivan Krezic, Lovorka Batelja Vuletic, Katarina Horvat Pavlov, Andrea Petrovic, Suncana Sikiric, Hrvoje Vranes, Andreja Prtoric, Helena Zizek, Tajana Durasin, Ivan Dobric, Mario Staresinic, Sanja Strbe, Mario Knezevic, Marija Sola, Antonio Kokot, Marko Sever, Eva Lovric, Anita Skrtic, Alenka Boban Blagaic, Predrag Sikiric

**Affiliations:** ^1^ Department of Pathology, School of Medicine, University of Zagreb, Zagreb, Croatia; ^2^ Department of Pharmacology, School of Medicine, University of Zagreb, Zagreb, Croatia; ^3^ Department of Surgery, School of Medicine, University of Zagreb, Zagreb, Croatia; ^4^ Department of Anatomy and Neuroscience, School of Medicine Osijek, University of Osijek, Osijek, Croatia

**Keywords:** stable gastric pentadecapeptide BPC 157, fistula, bleeding disorder, concept practical applicability, wound healing

## Abstract

**Significance:** The antiulcer peptide, stable gastric pentadecapeptide BPC 157 (previously employed in ulcerative colitis and multiple sclerosis trials, no reported toxicity (LD1 not achieved)), is reviewed, focusing on the particular skin wound therapy, incisional/excisional wound, deep burns, diabetic ulcers, and alkali burns, which may be generalized to the other tissues healing.

**Recent Advances:** BPC 157 has practical applicability (given alone, with the same dose range, and same equipotent routes of application, regardless the injury tested).

**Critical Issues:** By simultaneously curing cutaneous and other tissue wounds (colocutaneous, gastrocutaneous, esophagocutaneous, duodenocutaneous, vesicovaginal, and rectovaginal) in rats, the potency of BPC 157 is evident. Healing of the wounds is accomplished by resolution of vessel constriction, the primary platelet plug, the fibrin mesh which acts to stabilize the platelet plug, and resolution of the clot. Thereby, BPC 157 is effective in wound healing much like it is effective in counteracting bleeding disorders, produced by amputation, and/or anticoagulants application. Likewise, BPC 157 may prevent and/or attenuate or eliminate, thus, counteract both arterial and venous thrombosis. Then, confronted with obstructed vessels, there is circumvention of the occlusion, which may be the particular action of BPC 157 in ischemia/reperfusion.

**Future Directions:** BPC 157 rapidly increases various genes expression in rat excision skin wound. This would define the healing in the other tissues, that is, gastrointestinal tract, tendon, ligament, muscle, bone, nerve, spinal cord, cornea (maintained transparency), and blood vessels, seen with BPC 157 therapy.

## Scope and Significance

This stable gastric pentadecapeptide BPC 157 review (Seiwerth et al., 2018; Sikiric et al., 2020a; Sikiric et al., 2020b) is focused on the particular skin wound therapy, incisional/excisional wound (Seiwerth et al., 1997), deep burns (Mikus et al., 2001), diabetic ulcer (Tkalecevic et al., 2007), alkali burns (Huang et al., 2015), and healing of various other tissue types (Staresinic et al., 2003; Staresinic et al., 2006; Sever et al., 2009; Masnec et al., 2015; Becejac et al., 2018). The defensive system pertaining to BPC 157 beneficial activities was already appraised in several reviews (Sikiric et al., 1993; Sikiric et al., 2006; Sikiric et al., 2010; Sikiric et al., 2011; Sikiric et al., 2012; Sikiric et al., 2013; Seiwerth et al., 2014; Sikiric et al., 2014; Sikiric et al., 2016; Sikiric et al., 2017; Kang et al., 2018; Seiwerth et al., 2018; Sikiric et al., 2018; Gwyer et al., 2019; Park et al., 2020; Sikiric et al., 2020a; Sikiric et al., 2020b). A particular topic is its role in mediating Robert gastric cytoprotection and endothelial maintenance (Sikiric et al., 2010; Sikiric et al., 2011; Sikiric et al., 2017; Sikiric et al., 2018), as well as its therapeutic effect in the gastrointestinal tract (Sikiric et al., 2010; Sikiric et al., 2011; Sikiric et al., 2012; Sikiric et al., 2017; Seiwerth et al., 2018; Sikiric et al., 2018; Sikiric et al., 2020a; Sikiric et al., 2020b), additionally acting as membrane stabilizer (Park et al., 2020), with particular reference to ulcerative colitis (Sikiric et al., 2011). Recently, to approach particular skin wound therapy, we and others reviewed the significance of its beneficial effect on muscle, tendon, ligament, and bone injuries (Gwyer et al., 2019; Seiwerth et al., 2018).

## Translational Relevance

A special point confronts BPC 157 effectiveness with standard growth angiogenic factors, and their healing effects on the tendon, ligament, muscle, and bone lesions vs. their healing effects on gastrointestinal tract lesions (Seiwerth et al., 2018). Only BPC 157 has the same regimens, as used in the gastrointestinal healing studies, improving these lesions healing, accurately implementing its own healing angiogenic effect (Seiwerth et al., 2018). Additionally, we reviewed its particular effects, such as the non-steroidal anti-inflammatory drugs (NSAIDs) toxicity counteraction (Sikiric et al., 2013; Park et al., 2020), its relationship to the nitric oxide (NO)-system (Sikiric et al., 2014), and blood vessels (Sikiric et al., 2006; Seiwerth et al., 2014; Sikiric et al., 2018), and its role in the brain–gut and gut–brain axis (Sikiric et al., 2016), along with its CNS-disturbances therapy (Sikiric et al., 2016) and stress disorders (Sikiric et al., 2017; Sikiric et al., 2018).

## Clinical Relevance

As mentioned, the significance of its particular skin wound therapy was not especially reviewed. Namely, BPC 157 is always applied alone (i.e., its own effect ascribed only to the peptide (for review, see Sikiric et al., 1993; Sikiric et al., 2006; Sikiric et al., 2010; Sikiric et al., 2011; Sikiric et al., 2012; Sikiric et al., 2013; Seiwerth et al., 2014; Sikiric et al., 2014; Sikiric et al., 2016; Sikiric et al., 2017; Seiwerth et al., 2018; Kang et al., 2018; Sikiric et al., 2018; Gwyer et al., 2019; Sikiric et al., 2020a; Sikiric et al., 2020b). Unlike growth factors peptides, which need carrier(s) addition, and are rapidly degraded in human gastric juice, BPC 157, as an antiulcer peptide, is native and resistant to human gastric juice exciding one day period (Sikiric et al., 1993; Sikiric et al., 2006; Sikiric et al., 2010; Sikiric et al., 2011; Sikiric et al., 2012; Sikiric et al., 2013; Seiwerth et al., 2014; Sikiric et al., 2014; Sikiric et al., 2016; Sikiric et al., 2017; Kang et al., 2018; Seiwerth et al., 2018; Sikiric et al., 2018; Gwyer et al., 2019; Park et al., 2020; Sikiric et al., 2020a; Sikiric et al., 2020b). Therefore, BPC 157 practical applicability (given alone, with the same dose range, and the same equipotent routes of application, regardless of the injury tested) could be clearly generalized and used in the wound healing therapy.

## Background or Overview

Stable gastric pentadecapeptide BPC 157 is still far less investigated (Sikiric et al., 1993; Sikiric et al., 2006; Sikiric et al., 2010; Sikiric et al., 2011; Sikiric et al., 2012; Sikiric et al., 2013; Seiwerth et al., 2014; Sikiric et al., 2014; Sikiric et al., 2016; Sikiric et al., 2017; Kang et al., 2018; Seiwerth et al., 2018; Sikiric et al., 2018; Gwyer et al., 2019; Park et al., 2020; Sikiric et al., 2020a; Sikiric et al., 2020b) than the generally established angiogenic growth factors, epidermal growth factor (EGF), basic fibroblast growth factor (bFGF), and vascular endothelial growth factor (VEGF) (Tarnawski and Ahluwalia, 2012; Deng et al., 2013). Originally, BPC 157 appears as a cytoprotective antiulcer peptide, stable in human gastric juice, previously employed in ulcerative colitis clinical trials and now in those concerning multiple sclerosis (Sikiric et al., 1993; Sikiric et al., 2006; Sikiric et al., 2010; Sikiric et al., 2011; Sikiric et al., 2012; Sikiric et al., 2013; Seiwerth et al., 2014; Sikiric et al., 2014; Sikiric et al., 2016; Sikiric et al., 2017; Kang et al., 2018; Seiwerth et al., 2018; Sikiric et al., 2018; Park et al., 2020; Sikiric et al., 2020a; Sikiric et al., 2020b) without toxicity (lethal dose 1 (LD1) could be not obtained) (Sikiric et al., 1993; Sikiric et al., 2006; Sikiric et al., 2010; Sikiric et al., 2011; Sikiric et al., 2012; Sikiric et al., 2013; Seiwerth et al., 2014; Sikiric et al., 2014; Sikiric et al., 2016; Sikiric et al., 2017; Kang et al., 2018; Seiwerth et al., 2018; Sikiric et al., 2018; Gwyer et al., 2019; Park et al., 2020; Sikiric et al., 2020a; Sikiric et al., 2020b). As a cytoprotective agent, mediating Robert’s cytoprotection, it maintains endothelium integrity and has a particular angiomodulatory effect (Sikiric et al., 1993; Sikiric et al., 2006; Sikiric et al., 2010; Sikiric et al., 2011; Sikiric et al., 2012; Sikiric et al., 2013; Seiwerth et al., 2014; Sikiric et al., 2014; Sikiric et al., 2016; Sikiric et al., 2017; Kang et al., 2018; Seiwerth et al., 2018; Sikiric et al., 2018; Park et al., 2020; Sikiric et al., 2020a; Sikiric et al., 2020b) (in various wound models, VEGF, factor VIII, CD34 peak appear in the early interval, while later depressed (Brcic et al., 2009)). In the sponge assay, BPC 157 exhibits an angiogenic effect greater than the standard antiulcer agents (Sikiric et al., 1999a). Besides, when we consider the general wound principles in tissue damage as a dynamic equilibrium between negative and positive events (i.e., necrosis and possibly pus formation *vs.* activation of macrophages and fibroblasts), BPC 157 shows the particular full extent of its healing actions (Seiwerth et al., 1997). This includes in addition to the skin lesions, the concomitant lesions counteraction, that is, burn stress gastric lesions in severely burned mice (Mikus et al., 2001). As an extending point, there appears to be fistula healing (i.e., simultaneous healing of the skin and other tissues wounds) (Klicek et al., 2008; Skorjanec et al., 2009; Cesarec et al., 2013; Skorjanec et al., 2015; Baric et al., 2016; Grgic et al., 2016; Sikiric et al., 2020a). Illustratively, there are particular distinctions from the platelet-derived growth factor (PDGF-BB) activity (Seveljevic-Jaran et al., 2006; Tkalcevic et al., 2007). In contrast to PDGF-BB, in diabetic wounds, induced by alloxan application, BPC 157 largely promoted mature collagen in granulation tissue (Seveljevic-Jaran et al., 2006) (note, previously, BPC 157 healed alloxan-induced gastric lesions in rats (Petek et al., 1999)). In addition, BPC 157 has a particular beneficial effect when confronted with the major vessel occlusion (Duzel et al., 2017; Amic et al., 2018; Drmic et al., 2018; Vukojevic et al., 2018; Sever et al., 2019; Berkopic Cesar et al., 2020; Gojkovic et al., 2020; Kolovrat et al., 2020; Vukojevic et al., 2020). BPC 157 rapidly attenuates the major vessel occlusion severe consequences by rapidly activating collateral pathways occlusion (Duzel et al., 2017; Amic et al., 2018; Drmic et al., 2018; Vukojevic et al., 2018; Sever et al., 2019; Berkopic Cesar et al., 2020; Gojkovic et al., 2020; Kolovrat et al., 2020; Vukojevic et al., 2020). With permanent vessel obstruction, once the therapeutic effect begins, the beneficial action proceeds without further reappearance of the adverse effects of vessel obstruction (Duzel et al., 2017; Amic et al., 2018; Drmic et al., 2018; Vukojevic et al., 2018; Sever et al., 2019; Gojkovic et al., 2020; Kolovrat et al., 2020; Vukojevic et al., 2020). Likewise, reestablished blood flow may certainly contribute to the rapid recovery effect noted.

This particular balanced modulatory action, which rapidly appears, along with this pentadecapeptide’s particular characteristics, will be especially reviewed. It may be even more interesting and more effective than that of comparative standard agents (Sikiric et al., 1993; Sikiric et al., 2006; Sikiric et al., 2010; Sikiric et al., 2011; Sikiric et al., 2012; Sikiric et al., 2013; Seiwerth et al., 2014; Sikiric et al., 2014; Sikiric et al., 2016; Sikiric et al., 2017; Kang et al., 2018; Seiwerth et al., 2018; Sikiric et al., 2018; Gwyer et al., 2019; Park et al., 2020; Sikiric et al., 2020a; Sikiric et al., 2020b), the silver sulfadiazine cream (Mikus et al., 2001) or systemic corticosteroids (Sikiric et al., 2003), for possible wound healing therapy (i.e., incisional/excisional wound, deep burns, diabetic ulcer, and alkali burns) (Seiwerth et al., 1997; Mikus et al., 2001; Sikiric et al., 2003; Xue et al., 2004a; Bilic et al., 2005; Seveljevic-Jaran et al., 2006; Tkalcevic et al., 2007; Huang et al., 2015). Additionally, BPC 157 administration counteracted various free radical–induced lesions and increased free radical formation in other organs (Sikiric et al., 1993; Ilic et al., 2010; Belosic Halle et al., 2017; Duzel et al., 2017; Luetic et al., 2017; Amic et al., 2018; Drmic et al., 2018; Vukojevic et al., 2018; Sever et al., 2019; Sucic et al., 2019; Berkopic Cesar et al., 2020; Kolovrat et al., 2020). The carboxylic groups of the pentadecapeptide BPC 157 may contribute to its role as an antioxidant. The cumulative antioxidant activity could be very high with the reactivation of the carboxylic groups (e.g., glutathione or enzymes). Additionally, BPC 157 is present in most tissues (Seiwerth et al., 2018), where it can bind reactive free radicals and inactivate them at crucial positions not reachable by other antioxidants (Sikiric et al., 1993; Sikiric et al., 2006; Sikiric et al., 2010; Sikiric et al., 2011; Sikiric et al., 2012; Sikiric et al., 2013; Seiwerth et al., 2014; Sikiric et al., 2014; Sikiric et al., 2016; Sikiric et al., 2017; Kang et al., 2018; Seiwerth et al., 2018; Sikiric et al., 2018; Gwyer et al., 2019; Park et al., 2020; Sikiric et al., 2020a; Sikiric et al., 2020b).

### Skin Wounds

**FIGURE 1:**
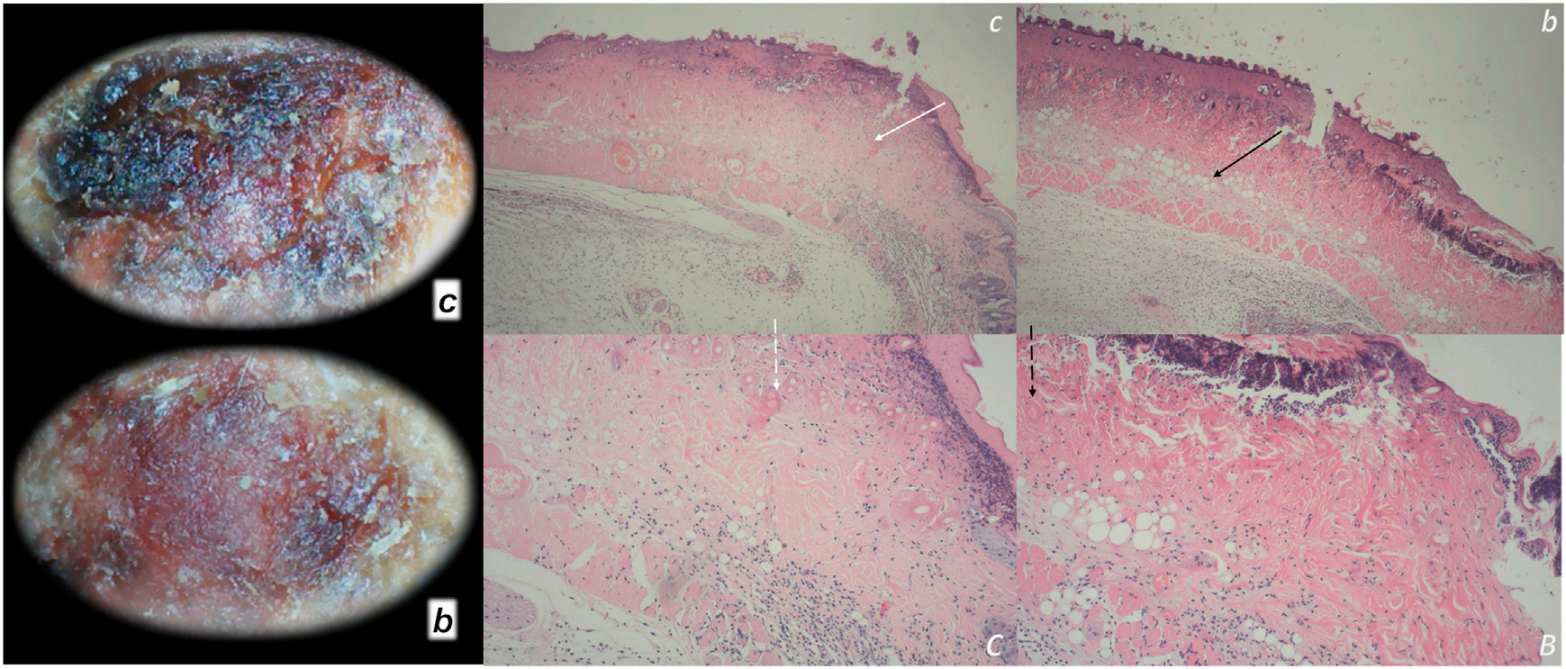
Burn skin lesions in mice and BPC 157 therapy effect. The effects of the gastric pentadecapeptide BPC 157 were investigated on deep partial skin thickness burns (1.5 × 1.5 cm) covering 20% of the total body area, when administered topically or systemically in burned mice (Mikus et al., 2001). Characteristic wound presentation at one week after injury, grossly, the poor healing in the untreated control or mice treated with vehicle only (*c (black letter)*) was completely reversed in BPC 157 cream–treated mice (*b (black letter)*) (1 μg/g neutral cream thin layer once time daily). Likewise, BPC 157 mice exhibited an increased breaking strength and relative elongation of burned skin and reduced water content in burned skin. Contrarily, silver sulfadiazine regimen did not achieve these healing effects. Microscopically **(lower)**, at the postinjury day 3, in control mice, the burned area exhibits severe edema in the dermis and subcutis as well as an exudate with abundant edematous fluid on the surface (white arrow). Coagulated blood vessel walls and a proportion of vessels with fibrin clots (dashed white arrow) (HE, x4 (*c*), x10 (*C*)). Contrarily, BPC 157 mice have much less pronounced edema (black arrow), weak cellular infiltrate, and exudate, and the blood vessels walls seem to be more preserved (dashed black arrow) and in more vessels, endothelial cell can be observed. Almost no arterial clots (HE, x4 (*b*), x10 (*B*)) were observed.

This particular balanced modulatory action was indicated in the initial manuscript (Seiwerth et al., 1997). This study already established that the combined triad of collagen-inflammatory cells–angiogenesis was accordingly upgraded, appearing at earlier intervals, more rapid, and advanced with BPC 157 therapy (Seiwerth et al., 1997). Quantitative analysis of collagen development as well as granulation tissue formation and angiogenesis was performed *in vivo* models, incisional skin wounds, colon–colon anastomoses, and synthetic sponge implants (Seiwerth et al., 1997). The applied rationale of skin and colon wounds (Seiwerth et al., 1997) covers the different healing patterns and dynamics of these organs related to their collagen structures (Eyre et al., 1984; Eckersley and Dudley, 1988; Hendriks and Mastboom, 1990). The noted wide effectiveness in all of these models postulates a quite general wound healing effect (Seiwerth et al., 1997). Thereby, the subsequent burn studies were on the burns covering 20% of total body area on the back of mice, open flame for 5 or 7 s (Mikus et al., 2001; Sikiric et al., 2003). The accelerated healing in burns of treated mice includes the activity of the pentadecapeptide BPC 157 (Figure 1), given locally (as a cream) or systemically (ip), on the inflammatory cells, edema, reticulin, collagen, necrosis, blood vessel formation, number of preserved follicles, re-epithelization, tensile breaking strength, and water content in burned skin. BPC 157 regimens also attenuated burn stress-gastric lesions (Mikus et al., 2001). Note, there is apparently smaller extent of the silver sulfadiazine cream effect (Mikus et al., 2001). Further positive outcome appears in the corticosteroid animals with severe burns (Mikus et al., 2001; Sikiric et al., 2003). With BPC 157 additional therapy, there are no characteristic corticosteroid adverse effects in the corticosteroid animals (Sikiric et al., 2003). As a beneficial property, inhibition of the inflammatory response did not impair wound healing and did not induce failed collagen synthesis (Mikus et al., 2001; Sikiric et al., 2003). In this, pentadecapeptide BPC 157 consistently (grossly, microscopically, and biomechanically) cured burn injuries, and counteracted corticosteroid (6α-methylprednisolone 1.0 or 10 mg/kg/day ip for 21 days)-induced impairment of burn healing (Mikus et al., 2001; Sikiric et al., 2003). With pentadecapeptide BPC 157, less edema and less inflammatory cells, re-epithelization, tensile breaking force, relative elongation of the burned part skin appear together, and inhibition of inflammation and beneficial effects. This chain of events fails to occur in the corticosteroids animals after initial inflammation inhibition (Sikiric et al., 2003). Even more importantly, BPC 157 accordingly inhibited corticosteroid-induced immunosuppression (Sikiric et al., 2003). *In vitro*, in comparison with control, healthy animals, the assessment of splenic cells (day 21) demonstrated that the 6α-methylprednisolone animals had declined reactivity to nitrogen, while the addition of BPC 157 (1 μg/g cream) returned cell reactivity to normal values (Mikus et al., 2001; Sikiric et al., 2003). A similar outcome appears in the CO_2_ laser injury on the dorsal skin mice (Bilic et al., 2005).

**FIGURE 2:**
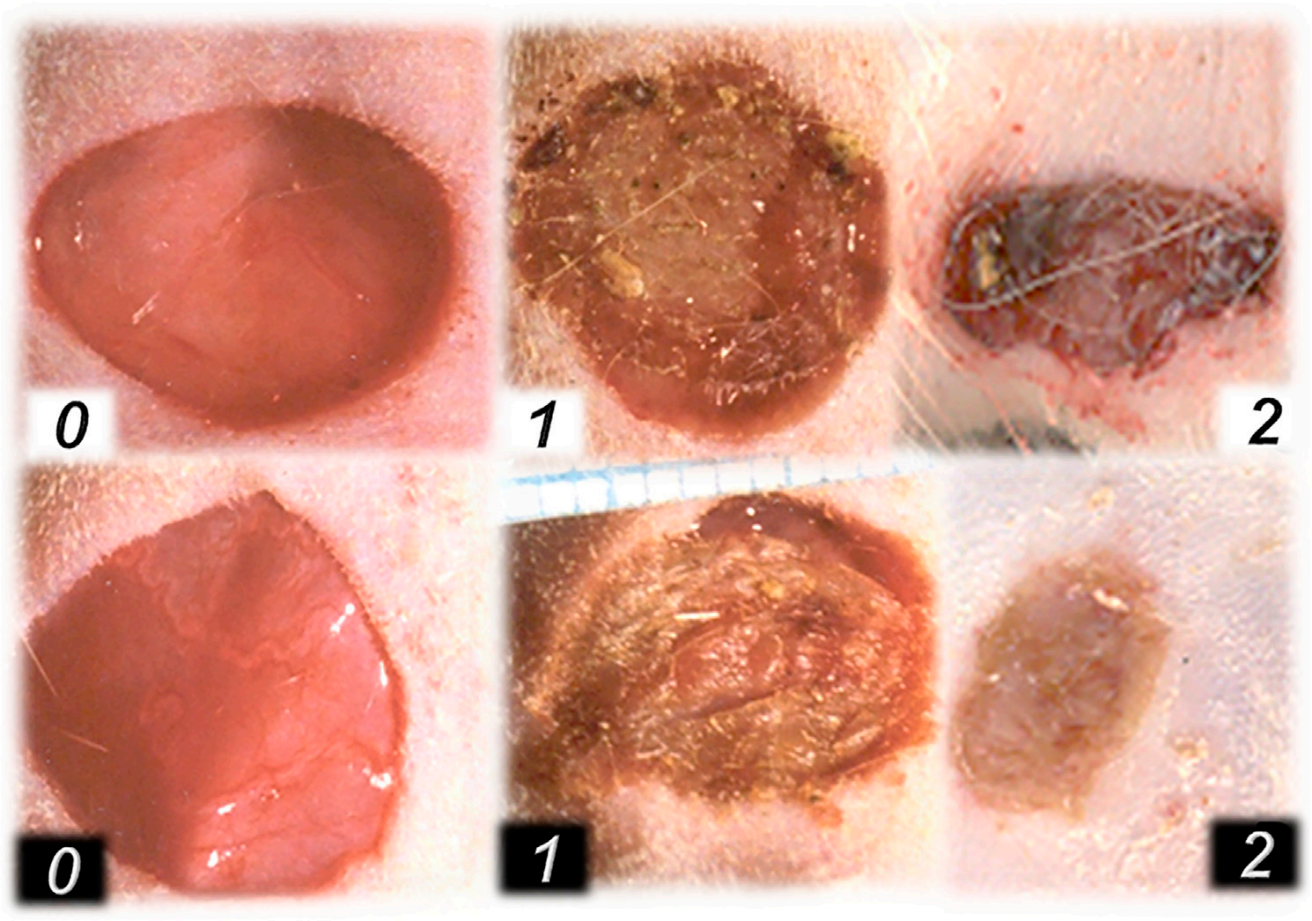
Excisional wound in diabetic rats (0, 1) and in rats with ligation of the right iliac artery and vein (2) **(upper)** and BPC 157 therapy **(lower)**. At 3 days before wounding, alloxan (300 mg/kg sc), thin layer of the BPC 157 cream (1 µg/1 g neutral cream) (white number), or neutral cream (black number) was immediately given upon wounding (*0, 0*). Advanced wound healing presentation at 24 h in BPC 157 rats (*1, white*), but not in controls (*1, black*). Likewise, advanced healing in the rats with ligated right iliac arteries and veins, when ingested BPC 157 through drinking water (10 μg/kg, 10 ng/kg, 0.16 μg/ml, 0.16 ng/ml, and 12 ml/rat/day) at postsurgery day 4 (*2, white*), but not in controls (*2, black*).

To find out a specific mechanism, a comparison with the becaplermin (recombinant human platelet–derived growth factor homodimer of B chains, PDGF-BB) was the focus of the two additional studies using excisional wounds in diabetic rats and mice (Figure 2) (Seveljevic-Jaran et al., 2006; Tkalcevic et al., 2007). Increased expression of the immediate response gene, early growth response gene-1 (egr-1), was shown in Caco-2 cells *in vitro* (Tkalcevic et al., 2007). These studies, even in diabetic conditions (Seveljevic-Jaran et al., 2006; Tkalcevic et al., 2007), revealed increased stimulation of early collagen organization in BPC 157 therapy. Importantly, BPC 157, but not PDGF-BB, stimulated earlier maturation of granulation tissue, soluble collagen concentration in the exudates (using sponge implantation), and organized collagen significantly more in wounds (i.e., on day 12 after daily treatment) (Tkalcevic et al., 2007). As a rationale, the study proposed the evidence that stimulation of the Caco-2 cells with 10–100 μM BPC 157 resulted in an earlier, reproducible stimulation of the expression of mRNA for egr-1, with a peak after 15 min. The peak expression of mRNA for the egr-1 co-repressor, nerve growth factor 1-A binding protein-2 (nab2), was observed 30 min after BPC 157 stimulation (Tkalcevic et al., 2007). This favors the possible controlling role of the BPC 157. Namely, we should consider egr-1 gene both positive and negative association. The beneficial significance of the egr-1 gene implies the healing process (i.e., trans-activation of many cognate target genes in healing tissue, including growth factors and cytokines (Braddock et al., 1999; Braddock, 2001), the transcription of other genes, including those for collagen II (α1) and PDGF (Alexander et al., 2002). Likewise, there is its negative association (i.e., elevated egr-1 levels associated with cardiovascular pathobiology (Khachigian, 2006) or cholestatic liver injury (Kim et al., 2006; Zhang et al., 2011). It may be that BPC 157 rapid activation of the egr-1 and its co-repressor, nab2, means an essential operating healing BPC 157 feedback axis controlling egr-1 levels along with nab2.

Finally, BPC 157 administration cured the alkali burn-induced skin injury (Huang et al., 2015). There were faster granulation tissue formation, re-epithelialization, dermal remodeling, and collagen deposition through extracellular signal–regulated kinases (ERK)1/2 signaling pathway as well as its downstream targets, including c-Fos, c-Jun, and egr-1. Likewise, there is greater proliferation of human umbilical vein endothelial cells (HUVECs), significantly promoted migration of HUVECs (transwell assay), and the upregulated expression of VEGF-a, and accelerated vascular tube formation *in vitro* (Huang et al., 2015).

Finally, this effect is apparently not species specific, and it was seen in the bigger animals as well. A similar finding (i.e., rehabilitation of skin wound and maturation of granulation tissue markedly promoted by BPC 157) was obtained in small-type pigs (Xue et al., 2004a). Note, the same group also reported a therapy with a prominent beneficial effect on various stomach lesions after administration of BPC 157 (Xue et al., 2004b).

### Practical Application as Support

The evidence reported for the BPC 157 is the effectiveness with peptide given alone (Sikiric et al., 1993; Sikiric et al., 2006; Sikiric et al., 2010; Sikiric et al., 2011; Sikiric et al., 2012; Sikiric et al., 2013; Seiwerth et al., 2014; Sikiric et al., 2014; Sikiric et al., 2016; Sikiric et al., 2017; Kang et al., 2018; Seiwerth et al., 2018; Sikiric et al., 2018; Gwyer et al., 2019; Park et al., 2020; Sikiric et al., 2020a; Sikiric et al., 2020b). Contrarily, when there is a peptide carrier, far more investigated and commonly implied angiogenic standard growth factors in the growth factor healing concept (Tarnawski and Ahluwalia, 2012; Deng et al., 2013) have several limitations (i.e., local application, carrier addition, and weak and uncertain peptide activity of its own (Seiwerth et al., 2018)), delaying practical realization of the growth factor healing concept.

Illustratively, in the mentioned laser-wound studies, human albumin solder supplemented with transforming growth factor (TGF)-β1 only, but not with HB-EGF or bFGF, increased the early postoperative strength of laser-welded wounds (Poppas et al., 1996), even though the superpulsed CO_2_ laser enhanced fibroblast replication and appeared to stimulate bFGF and inhibit TGF-β1 secretion (Nowak et al., 2000). Likewise, an essential problem with regard to the active (and effective) peptide is the continuous search for new delivery systems and new carriers, and new carriers and delivery systems together, with all standard angiogenic growth factors to improve their efficacy, although they are very extensive and highly sophisticated (for review, see Saghazadeh et al., 2018; Jee et al., 2019). Obviously, it may be unclear which one of the parts of peptide + carrier complex would be responsible for the activity (for review, see Urist, 1996). Thereby, this pitfall (i.e., the more carriers, the less own peptide activity) is commonly not considered (Saghazadeh et al., 2018; Jee et al., 2019), despite the fact it is recognized (Urist, 1996), and eventually jeopardizes the conclusions about these peptide activities and significance (Tarnawski and Ahluwalia, 2012; Deng et al., 2013). Obviously, the effects of one growth factor could be not unified when obtained with addition (and help) of different carriers (Seiwerth et al., 2018), and regardless given highly sophisticated evidence (Tarnawski and Ahluwalia, 2012; Deng et al., 2013), they could make the erroneous conclusions. Thus, it may be that ignoring these attribution problems jeopardizes the current growth factor healing concept (Tarnawski and Ahluwalia, 2012; Deng et al., 2013) since the needed certainty that the full healing evidence was correctly ascribed to the given peptid, is obviously lacking. For example, FGF studies in 1990s (Damien et al., 1993; Thorén and Aspenberg, 1993; Siegall et al., 1994; Walter et al., 1996; Wang and Aspenberg, 1996; Yu et al., 1998; Tabata et al., 1999) are illustrative for the pertinence of the poorly resolved problem, illustrative for the diversity of the carriers (i.e., hyaluronate gel carrier (Wang and Aspengberg, 1996), alginate/heparin–sacharose microspheres and films (Yu et al., 1998), cellulose gel (Thorén and Aspenberg, 1993), defective form of *Pseudomonas* exotoxin (Siegall et al., 1994), fibrin adhesive carrier (Walter et al., 1996), biodegredable hydrogen gelatin (Tabata et al., 1999), natural coral, and collagen (Damien et al., 1993)). Obviously, as indicated (Sikiric et al., 2018), there is an inescapable diversity of the carriers and thereby, an evident diversity of the obtained beneficial effects, and disable conclusion. Likewise, for bone morphogenic proteins (BMPs), at that time, besides bone matrix, the following biomaterials have been tested as carriers: calcium phosphate, collagen, gelatin, and starch (Miyamoto et al., 1992; Gao et al., 1993; Katoh et al., 1993; Kawai et al., 1993; Cook et al., 1994; Kato et al., 1995; Kawakami et al., 1997). Some carriers (true ceramics and pure titanium) remain in the bone tissue, whereas others (collagen and synthetic polymers) are absorbed. Some are absorbed so quickly that there is no enough time for a population of host cells to gather (Kawakami et al., 1997). Finally, the statement of Marshall Urist, the BMPs concept founder, about the mechanism of the release from BMP delivery system to mesenchymal cell receptor mechanisms as obscure and under intensive investigation in academic and industrial laboratories (Urist, 1996) is true also nowadays, and it can be generally applied to any of the peptide + carrier complexes.

Contrarily, BPC 157 has a general healing argument as general application protocol. Namely, it is always applied alone (Sikiric et al., 1993; Sikiric et al., 2006; Sikiric et al., 2010; Sikiric et al., 2011; Sikiric et al., 2012; Sikiric et al., 2013; Seiwerth et al., 2014; Sikiric et al., 2014; Sikiric et al., 2016; Sikiric et al., 2017; Kang et al., 2018; Seiwerth et al., 2018; Sikiric et al., 2018; Gwyer et al., 2019; Park et al., 2020; Sikiric et al., 2020a; Sikiric et al., 2020b). Unlike other growth factors, which need carrier(s) addition, and are rapidly destroyed in human gastric juice, BPC 157 is native and stable in human gastric juice for more than 24 h (Sikiric et al., 2018; Sikiric et al., 2020a; Sikiric et al., 2020b). As BPC 157 counteracts lesions in the whole gastrointestinal tract produced by various noxious procedures (Sikiric et al., 1993; Sikiric et al., 2006; Sikiric et al., 2010; Sikiric et al., 2011; Sikiric et al., 2012; Sikiric et al., 2013; Seiwerth et al., 2014; Sikiric et al., 2014; Sikiric et al., 2016; Sikiric et al., 2017; Kang et al., 2018; Seiwerth et al., 2018; Sikiric et al., 2018; Gwyer et al., 2019; Park et al., 2020; Sikiric et al., 2020a; Sikiric et al., 2020b), it may be indeed that it may endogenously maintain gastrointestinal mucosa integrity (Sikiric et al., 1993; Sikiric et al., 2006; Sikiric et al., 2010; Sikiric et al., 2011; Sikiric et al., 2012; Sikiric et al., 2013; Seiwerth et al., 2014; Sikiric et al., 2014; Sikiric et al., 2016; Sikiric et al., 2017; Kang et al., 2018; Seiwerth et al., 2018; Sikiric et al., 2018; Gwyer et al., 2019; Park et al., 2020; Sikiric et al., 2020a; Sikiric et al., 2020b). Consequently, BPC 157 may have beneficial activity with the same dose range, and same equipotent routes of application, regardless of injury tested (Sikiric et al., 1993; Sikiric et al., 2006; Sikiric et al., 2010; Sikiric et al., 2011; Sikiric et al., 2012; Sikiric et al., 2013; Seiwerth et al., 2014; Sikiric et al., 2014; Sikiric et al., 2016; Sikiric et al., 2017; Kang et al., 2018; Seiwerth et al., 2018; Sikiric et al., 2018; Gwyer et al., 2019; Park et al., 2020; Sikiric et al., 2020a; Sikiric et al., 2020b). Therefore, this could be clearly generalized, and thus, its own effect unmistakably ascribed only to the given peptide (for review, see Seiwerth et al., 2018). Besides, as indicated specifically in skin wound healing (Tkalcevic et al., 2007), BPC 157 half-life is long enough to exert a therapeutically stimulating effect on connective tissue growth (Tkalcevic et al., 2007). In sponge exudates, it remains active at the site of wounds for several hours (Tkalcevic et al., 2007).

The generalization of the skin wound healing therapy (Seiwerth et al., 1997; Mikus et al., 2001; Sikiric et al., 2003; Xue et al., 2004a; Bilic et al., 2005; Seveljevic-Jaran et al., 2006; Tkalcevic et al., 2007; Huang et al., 2015) as a particular point, will be illustrated with the subsequent successful healing of the various fistulas (thereby, the simultaneous healing of the different tissues) (Klicek et al., 2008; Skorjanec et al., 2009; Cesarec et al., 2013; Skorjanec et al., 2015; Baric et al., 2016; Grgic et al., 2016; Sikiric et al., 2020a) (see *Fistula Healing as Support*). Next, its particular therapeutic result on wound healing is decreased bleeding (Stupnisek et al., 2012; Stupnisek et al., 2015), and during wounding and recovery, all four major events in clot formation and dissolution were accomplished (Stupnisek et al., 2012). This was illustrated in the therapy of the bleeding disorders (Stupnisek et al., 2012; Stupnisek et al., 2015) (see *Therapy of Bleeding Disorders as Support*).

### Fistula Healing as Support

BPC 157 application successfully cured various fistulas (Sikiric et al., 2020a). Anastomoses between two defects in the corresponding tissues (i.e., in esophagus and skin (Cesarec et al., 2013), stomach and skin (Skorjanec et al., 2009), duodenum and skin (Skorjanec et al., 2015), colon and skin (Klicek et al., 2008), colon and bladder (Grgic et al., 2016), and rectum and vagina (Baric et al., 2016)) depict the various fistulas (Sikiric et al., 2020a). Likewise, these defects of the controlled size may fairly illustrate accelerated or enabled healing (i. e., closure) and wound/gastrointestinal ulcer relation (Sikiric et al., 2020a).

Of note, the methodology of this healing evidence (Klicek et al., 2008; Skorjanec et al., 2009; Cesarec et al., 2013; Skorjanec et al., 2015; Baric et al., 2016; Grgic et al., 2016; Sikiric et al., 2020a) may contrast with the miscellaneous underlying fistula causes, various origin and location and different occurrences (Orangio, 2010; Visschers et al., 2012; Tonolini and Magistrelli, 2017). Likewise, this approach may contrast with therapeutic tactics for fistulas, which depend on their location and severity of occurrence (Orangio, 2010; Visschers et al., 2012; Tonolini and Magistrelli, 2017). Also, it opposes the nowadays aggressive wound management if it considers in fistulas healing mostly local skin protection (Orangio, 2010; Visschers et al., 2012; Tonolini and Magistrelli, 2017). On the other hand, this healing evidence as such (Klicek et al., 2008; Skorjanec et al., 2009; Cesarec et al., 2013; Skorjanec et al., 2015; Baric et al., 2016; Grgic et al., 2016; Sikiric et al., 2020a) may accommodate and resolve the mentioned fistula diversity (Orangio, 2010; Visschers et al., 2012; Tonolini and Magistrelli, 2017) as a common healing denominator providing simultaneous healing of different tissues.

**FIGURE 3:**
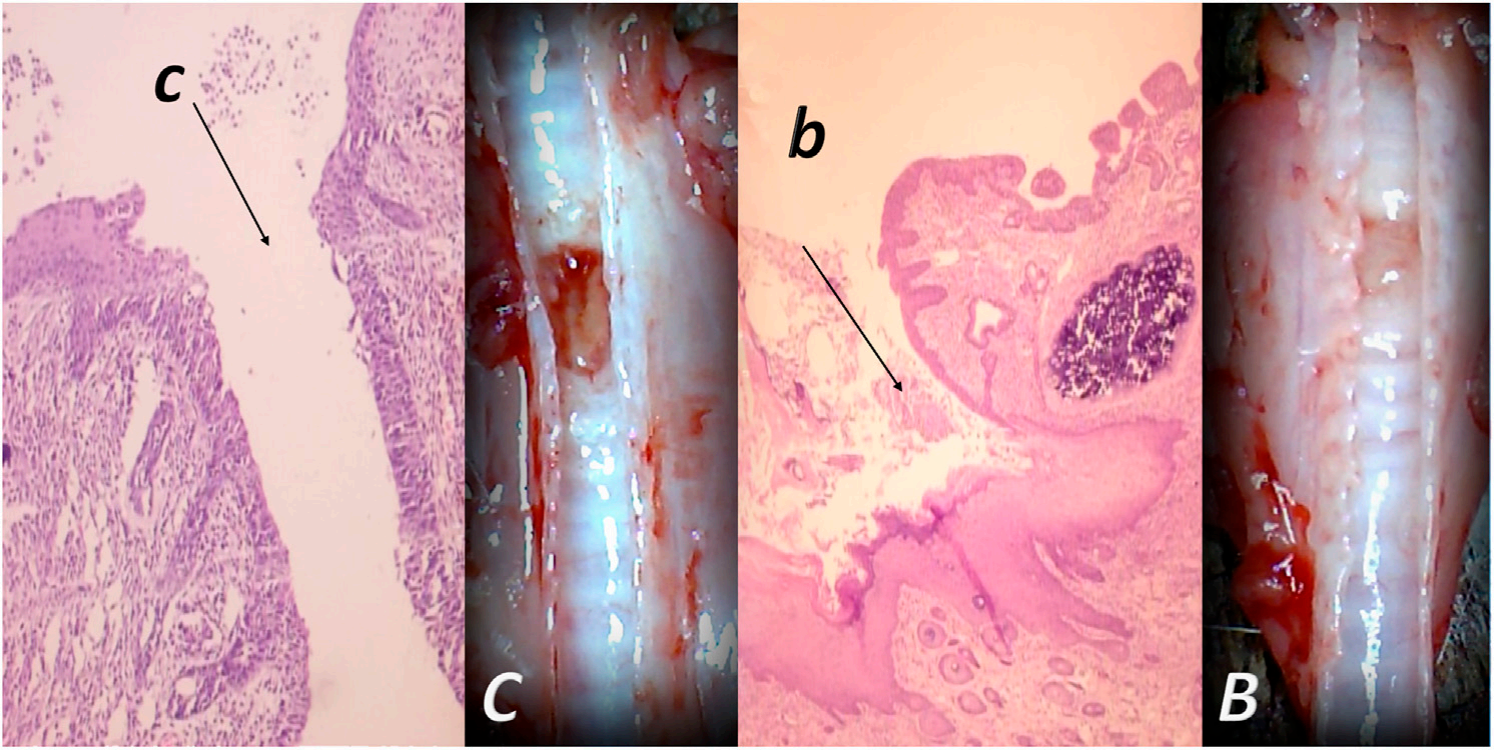
Tracheocutaneous fistulas and BPC 157 therapy. After injury induction, BPC 157 dissolved in saline (10 μg, 10 ng/kg body weight) given per-orally in drinking water till the sacrifice (0.16 μg/ml, 0.16 ng/ml, and 12 ml/day/rat). At postsurgery day 7, fistula closure, closed tracheal defect, and closed skin defect (*B, b*) (arrow) were observed in BPC 157 rats. Contrarily, in controls, fistula remained open, fistulous channel was formed in the skin, and open tracheal defects were observed (*C, c*) (arrow) (HE, x4).

**FIGURE 4:**
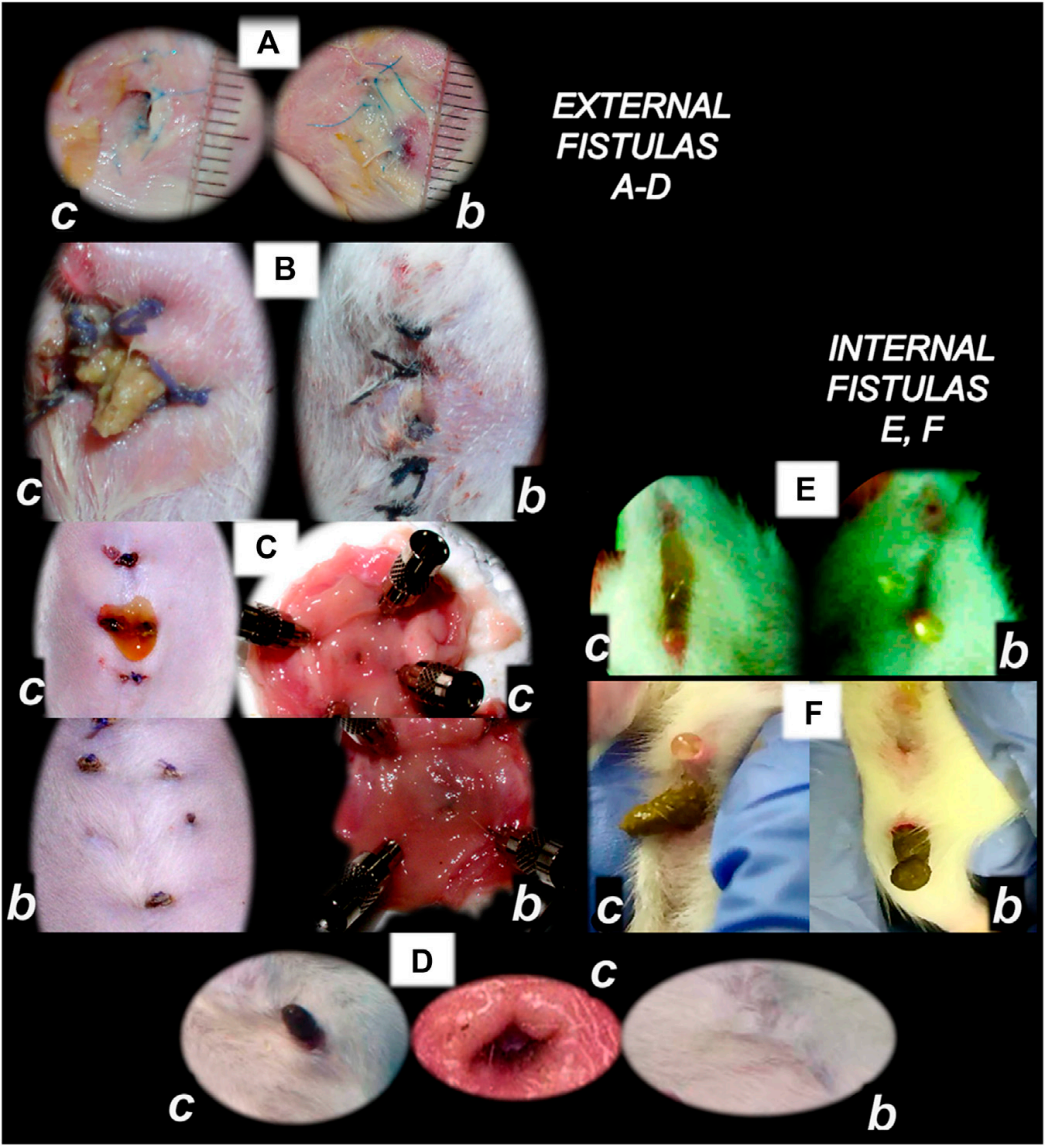
BPC 157 and fistulas closing. External **(A–D)** and internal **(E, F)** fistulas. External fistulas. **(A)** Persistent esophagocutaneous fistula and BPC 157 therapy effect. BPC 157 was given per-orally, in drinking water (10 μg/kg, 10 ng/kg, i.e., 0.16 μg/ml, 0.16 ng/ml, 12 ml/rat/day) until sacrifice, or intraperitoneally (10 μg/kg, 10 ng/kg) with first application at 30 min after surgery, last at 24 h before sacrifice. To establish NO-system involvement, L-NAME (5 mg/kg i.p.) (worsening) and/or L-arginine (100 mg/kg i.p.) (beneficial effect) were given alone or together; first application at 30 min after surgery, last at 24 h before sacrifice. BPC 157 (10 μg/kg, i.p. or p.o.) given with L-NAME (5 mg/kg i.p.) and/or L-arginine (100 mg/kg i.p.) and it maintained its original beneficial effect. A closely interrelated process of unhealed skin, esophageal defects, unhealed fistulas (upregulated eNOS, iNOS, and COX2 mRNA levels), usually lethal, particularly NO-system–related and therapy dependent, illustrate a largely open skin defect in controls at day 3 (*c*) and a closed skin defect in BPC 157 rats (*b*) (Ceserec et al., 2013). **(B**) Initial presentation of the persistent gastrocutaneous fistula and BPC 157 therapy effect. Huge gastrocutaneous fistula leaking at 1st postoperative day in control rats (*c*) and dried fistula without any leakage in BPC 157 rats (*b* (Skorjanec et al., 2009). The rats received pentadecapeptide BPC 157 (0.16 μg/ml) in drinking water (12 ml/rat) until sacrifice or drinking water only. A comparative study of the BPC 157 beneficial effect was done with intraperitoneal application, once daily, intraperitoneally (per kg body weight) 10 μg, 10 ng, or 10 pg BPC 157, while standard agents 10 mg atropine, 50 mg ranitidine, and 50 mg omeprazole provide only a weak effect. 6-alpha-methylprednisolone (1 mg/kg intraperitoneally, once daily) was given alone which produced a considerable worsening and was completely eliminated with coadministration of BPC 157 10 μg/kg intraperitoneally. **(C)** Persistent duodenocutaneous fistula and BPC 157 therapy effect. BPC 157 was given per-orally, in drinking water (10 μg/kg, 10 ng/kg, i.e., 0.16 μg/ml, 0.16 ng/ml, and 12 ml/rat/day) till sacrifice, or alternatively, 10 μg/kg and 10 ng/kg intraperitoneally; first application at 30 min after surgery, last at 24 h before sacrifice. To establish a connection with the NO-system, l-NAME (5 mg/kg intraperitoneally) (worsening) and/or L-arginine (100 mg/kg intraperitoneally) (beneficial effect) were given alone or together; first application at 30 min after surgery, last at 24 h before sacrifice. BPC 157 10 μg/kg, intraperitoneally or per-orally, was given with l-NAME (5 mg/kg intraperitoneally) and/or L-arginine (100 mg/kg intraperitoneally) and it maintained its original beneficial effect. Controls simultaneously received an equivolume of saline (5.0 ml/kg intraperitoneally) or water only. Duodenal fistula leaking through skin defect and still open duodenal defect at 2 weeks following fistula creation by anastomosis between the skin and duodenum defect (*c*). Closed both skin and duodenal defect in BPC 157 rats (fistula closed, *b*) (Skorjanec et al., 2015). **(D)** Persistent colocutaneous fistula and BPC 157 therapy effect. BPC 157 accelerated parenterally or per-orally the healing of colonic and skin defect, leading to the suitable closure of the fistula, macro/microscopically, biomechanically, and functionally (larger water volume sustained without fistula leaking) (Klicek et al., 2013). In anesthetized rats, we created the colocutaneous fistula at 5 cm from the anus, colon defect of 5 mm, and skin defect of 5 mm. The rats received pentadecapeptide BPC 157 (0.16 μg/ml) or nothing in the drinking water (12.0 ml/rat) until the sacrifice or once daily, intraperitoneally BPC 157 10.0 μg/kg, 10.0 ng/kg, or saline (5.0 ml/kg b.w.); first application at 30 min after surgery, final 24 h before sacrifice. For comparison, sulfasalazine (50 mg/kg intraperitoneally, once daily) (moderately effective) or 6-α-methylprednisolone (1.0 mg/kg intraperitoneally, once daily) (aggravation) was given. To establish connection with the NO-system, L-NAME (5.0 mg/kg) (worsening) and L-arginine (200.0 mg/kg) (effective only with blunted NO-synthesis, but not alone) were given intraperitoneally alone or in combination (d-arginine 200.0 mg/kg was not effective, data not shown). BPC 157 given with NO-agents, which maintained its original effect. Initial colon defect presentation at 4 weeks following fistula creation by anastomosis between the skin and colon defect (c, middle). Presentation after next 2 weeks (*c, b*): in control rats, drinking water was continuously given (12 ml/day/rat) (defecation through fistula, *c*) and in BPC 157 rats, BPC 157 (10 μg/kg/day) was given in drinking water (0.16 μg/ml/day/rat) (fistula closed, *b*). Internal fistulas. **(E)** Persistent colovesical fistula and BPC 157 therapy effect. With internal fistulas in the colon and the bladder, with BPC 157 therapy, the colon and bladder defects showed simultaneous healing effects, including closing of the colovesical fistula in a matching healing process (Grgic et al., 2016). BPC 157 was given per-orally in drinking water (10 μg/kg, 12 ml/rat/day) until sacrifice, or 10 μg/kg or 10 ng/kg was given intraperitoneally once daily, with the first application at 30 min after surgery and the last application at 24 h before sacrifice. The controls simultaneously received an equivolume of saline (5.0 ml/kg ip) or water only (12 ml/rat/day). At postoperative day 28, voiding through fistula in controls (fecaluria) (*c*) and a normal voiding in BPC 157-rats (*b*). **(F)** Persistent rectovaginal fistula and BPC 157 therapy effect. We suggest BPC 157 healing of the rats’ rectovaginal fistulas (since spontaneous only poor healing as those in humans) as a realization of the internal fistula-healing concept, an efficient “wound-healing capability” as the therapy of the complicated internal fistula healing. BPC 157 was given per-orally, in drinking water (10 μg/kg or 10 ng/kg, 0.16 μg/ml, or 0.16 ng/ml 12 ml/rat/day) till sacrifice, or alternatively, 10 μg/kg and 10 ng/kg intraperitoneally once daily; first application at 30 min after surgery, last at 24 h before sacrifice. Controls simultaneously received an equivolume of saline (5.0 ml/kg ip) or water only (12 ml/rat/day). At postoperative day 21, defecation through vagina in controls (*c*) and a normal defecation in BPC 157 rats (*b*) (Baric et al., 2016).

Consequently, we hold fistulas commonality (Klicek et al., 2008; Skorjanec et al., 2009; Cesarec et al., 2013; Skorjanec et al., 2015; Baric et al., 2016; Grgic et al., 2016; Sikiric et al., 2020a) and thereby, the revealing common resolution (Sikiric et al., 2020a). There are two different tissues simultaneously affected and a healing process that would organize synchronized healing (Sikiric et al., 2020a). Thus, the main implication of the fistula healing model as wounds with abnormal connections is the verification of the effectiveness of the skin wound healing as understood with BPC 157 effects in the skin wound models (Seiwerth et al., 1997; Mikus et al., 2001; Sikiric et al., 2003; Xue et al., 2004a; Bilic et al., 2005; Seveljevic-Jaran et al., 2006; Tkalcevic et al., 2007; Huang et al., 2015). That healing may be smoothly generalized simultaneously to the other tissues (Figure 3), and this particular balanced modulatory action may have the general significance, with the eventual healing of the affected tissues (Figure 4) (Klicek et al., 2008; Skorjanec et al., 2009; Cesarec et al., 2013; Skorjanec et al., 2015; Baric et al., 2016; Grgic et al., 2016; Sikiric et al., 2020a). Otherwise, the healing of the different tissues, which are normally not connected, would hardly provide a simultaneous wound healing effect, particularly in the most critical circumstances (Klicek et al., 2008; Skorjanec et al., 2009; Cesarec et al., 2013; Skorjanec et al., 2015; Baric et al., 2016; Grgic et al., 2016; Sikiric et al., 2020a). Note that due to the relative small size of the rats, rat fistulas regularly appeared as large and complex, and thereby, in this respect, corresponded to the worst presentation in the patients (Klicek et al., 2008; Skorjanec et al., 2009; Cesarec et al., 2013; Skorjanec et al., 2015; Baric et al., 2016; Grgic et al., 2016; Sikiric et al., 2020a). Harmful disturbances and poor healing of rectovaginal fistulas in patients (Baric et al., 2016) may occur also in rats (i.e., fecal leaking through the vagina). Rats could not endure more than 4 days with esophagocutaneous fistulas (Cesarec et al., 2013). However, with BPC 157 therapy, both skin and esophageal defect may be closed without mortality (Cesarec et al., 2013). Thereby, that healing in rat fistulas in these experiments should be highly relevant (Klicek et al., 2008; Skorjanec et al., 2009; Cesarec et al., 2013; Skorjanec et al., 2015; Baric et al., 2016; Grgic et al., 2016; Sikiric et al., 2020a). Obviously, a distinctive correlation follows with the major systems generally involved in the healing processes (Sikiric et al., 2020a).

Basically, closure of fistulas may be a measure of particular agent’s capacity to cure, at the same time, the skin wound and other corresponding tissues’ wound (Klicek et al., 2008; Skorjanec et al., 2009; Cesarec et al., 2013; Skorjanec et al., 2015; Baric et al., 2016; Grgic et al., 2016; Sikiric et al., 2020a). The healing of the skin and colon defect, as colocutaneous fistulas, stable gastric pentadecapeptide BPC 157, and the therapy regimens (per-oral, in drinking water; or intraperitoneal (once time daily) for 28 days) may serve as a prototype (for review, see Sikiric et al., 1993; Sikiric et al., 2006; Sikiric et al., 2010; Sikiric et al., 2011; Sikiric et al., 2012; Sikiric et al., 2013; Seiwerth et al., 2014; Sikiric et al., 2014; Sikiric et al., 2016; Sikiric et al., 2017; Kang et al., 2018; Seiwerth et al., 2018; Sikiric et al., 2018; Gwyer et al., 2019; Sikiric et al., 2020a; Sikiric et al., 2020b).

Also, a similar protocol was successful in the therapy of the other fistulas, that is, gastrocutaneous (Skorjanec et al., 2009), esophagocutaneous (Cesarec et al., 2013), duodenocutaneous (Skorjanec et al., 2015), vesicovaginal (Grgic et al., 2016), and rectovaginal (Baric et al., 2016) in rats. Noteworthy, as shown in separate studies, BPC 157 counteracts the known lesions in the skin (Seiwerth et al., 1997; Mikus et al., 2001; Sikiric et al., 2003; Xue et al., 2004a; Bilic et al., 2005; Seveljevic-Jaran et al., 2006; Tkalcevic et al., 2007; Huang et al., 2015), stomach (Sikiric et al., 1997a; Petek et al., 1999; Mikus et al., 2001; Xue et al., 2004b; Becejac et al., 2018; Ilic et al., 2009; Ilic et al., 2011a), duodenum (Sikiric et al., 1994; Sikiric et al., 1997b; Sikiric et al., 2001; Bedekovic et al., 2003; Amic et al., 2018), esophagus (Sikiric et al., 1999b; Petrovic et al., 2006; Dobric et al., 2007; Djakovic et al., 2016), colon (Sikiric et al., 2001; Klicek et al., 2013), rectum (Sikiric et al., 2001; Klicek et al., 2013), bladder (Sucic et al., 2019), and vagina (Jandric et al., 2013), whereas the fistula studies show an additional combining healing effect, providing different combinations of the lesions that were simultaneously included (Figures 3, 4), thereby a proof of the concept for a quite general healing effect (Klicek et al., 2008; Skorjanec et al., 2009; Cesarec et al., 2013; Skorjanec et al., 2015; Baric et al., 2016; Grgic et al., 2016; Sikiric et al., 2020a). A particular relationship was established with the NO-system, and the advantage of the BPC 157 over the corresponding standard agents (i.e., corticosteroids, sulfasalazine, H2 blockers, anticholinergics, and proton pump inhibitors) which showed only weak, if any, effect on these fistulas closing (Klicek et al., 2008; Skorjanec et al., 2009; Cesarec et al., 2013; Skorjanec et al., 2015; Baric et al., 2016; Grgic et al., 2016; Sikiric et al., 2020a).

Gastrocutaneous (Skorjanec et al., 2009) and/or duodenocutaneous (Skorjanec et al., 2015) fistulas, as persistent lesions, are another instructive prototype of the “two-ways” model. Often, the peptic ulcers’ inability to heal is taken as analogous to the chronic skin wound inability to heal; thus, resistant peptic ulcers equal to resistant chronic skin ulcers, both unable to heal (Skorjanec et al., 2009). Contrary to the use of the gastrocutaneous fistula for the secretory studies only (Skorjanec et al., 2009), the wound/gastrointestinal ulcer relation of these gastrocutaneous fistulas healing exemplifies the reported particular “self-controlling healing system” (Skorjanec et al., 2009). Only the simultaneous healing of the skin defect and the stomach defect would lead to the fistula closure (Skorjanec et al., 2009). Since classic models are not combined, gastric/duodenal ulcer models (Selye and Szabo, 1973; Robert, 1979; Okabe and Amagase, 2005) would define agents’ action. Likewise, skin defect models (Rantfors and Cassuto, 2003; Numata et al., 2006; Rao et al., 2007) would explain agents’ action (“one-way” model). Thus, there is a practical advantage of the composed effects of the administered agents, and the gastrocutaneous fistulas as a combined (“two-ways”) model. For example, for mutual definition (model → agent; agent → model), there are prostaglandins analogues—ethanol model (Robert, 1979) relationships, NSAIDs—acetic acid model (Okabe and Amagase, 2005) relationships, dopamine agonists—cysteamine model (Selye and Szabo, 1973) relationships, and H_2_ blockers—cysteamine model (Selye and Szabo, 1973) relationships. Thereby, studies of gastrocutaneous fistulas (Skorjanec et al., 2009) may resolve in the wound/gastrointestinal ulcer relation. This may be the improvement or aggravation that tested agents can exhibit of healing. This may be healing of the skin or gastric wound, or both, or neither of them, simultaneously or not. This may identify the so-called parallel or non-parallel healing actions, with the final end result, positive (fistula closing) or negative (fistula remains open) (Skorjanec et al., 2009). Besides, BPC 157 promptly ameliorates both skin and stomach mucosa healing and induces closure of fistulas, with no leakage after up to 20 ml water intragastrically, including also counteraction of 6-alpha-methylprednisolone aggravation (Skorjanec et al., 2009).

### Therapy of Bleeding Disorders as Support

**FIGURE 5:**
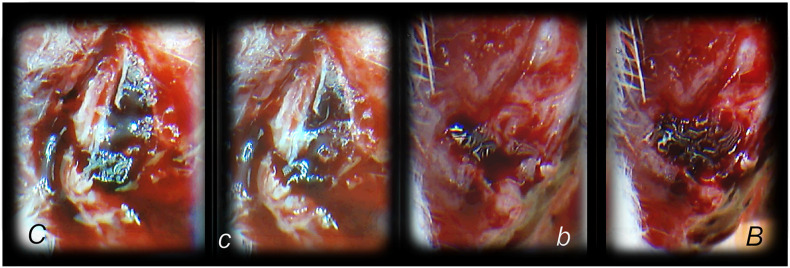
Hematoma formation following tibial diaphysis fracture and BPC 157 therapy effect, in analogy with accomplished all four events (vessel constriction, the primary platelet plug, the secondary plug, and resolution of the clot) that occur in a set order following the loss of vascular integrity (Stupnisek et al., 2012) involved in the wound healing. We suggest BPC 157 healing starting with a rapid formation of the adequate hematoma as a connective scaffold between the stumps. BPC 157 (10 ng/kg) was given as a 1-ml bath to the injury, immediately after injury induction. Controls simultaneously received an equal of saline as a bath to the injury. At 1 min after application, hematoma within fracture gap (*b*) further progressed at 2 min (*B*) in BPC 157 rats. Diffuse bleeding in controls at 1 min (*c*) and 2 min (*C*), weak hematoma formed outside of the fracture gap.

**FIGURE 6:**
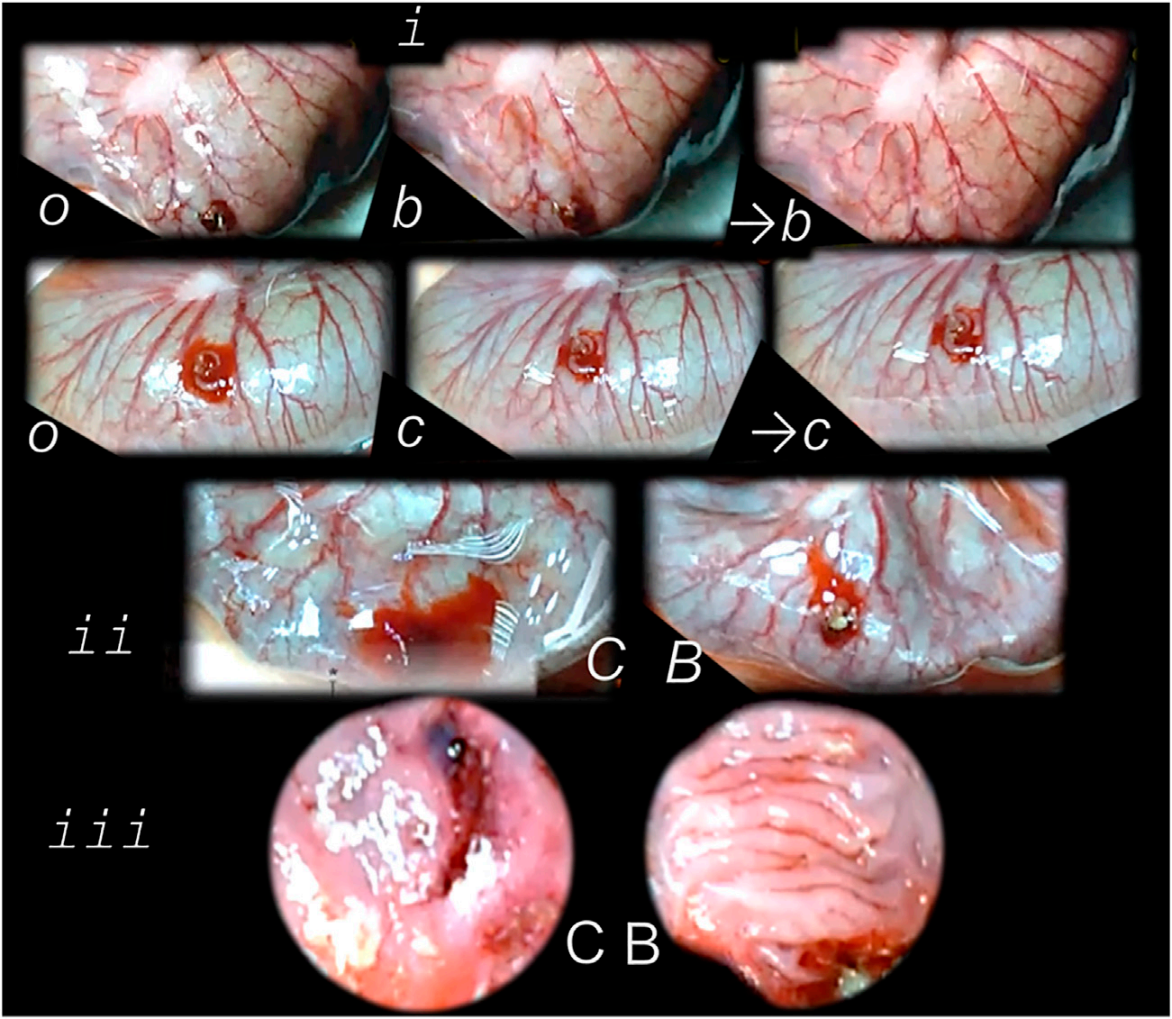
Perforated cecum defect and BPC 157 therapy effect. *i*. Presentation of the perforated cecum defect (*o*-immediately after perforation, before therapy) as an illustration of the rapid healing effect immediately after wounding, with vessels “running” toward the defect augmented by BPC 157 bath application (10 μg/kg) (USB microcamera). *b—*vessels recruitment presentation immediately under the immersion of the BPC 157 bath, which had been applied at the cecum, and presentation immediately thereafter *→b*) with corresponding controls (saline bath 1 ml/rat) presentation *c, →c*). *ii*. Resultant bleeding from the perforated defect (*C* (controls), decreased in BPC 157 rats (B). *iii*. Final failure of the perforated defect healing in controls (C) (postinjury day 7) and completely healed defect in BPC 157 rats (B). This beneficial effect goes along with counteraction of the worsening effect of both NOS-blocker L-NAME (5 mg/kg), or NOS substrate L-arginine (100 mg/kg) (directly applied to the perforated cecum, alone or combined, and spread through the abdominal cavity), and normalization of the increased MDA- and NO-values in the cecum (Drmic et al., 2018).

**FIGURE 7:**
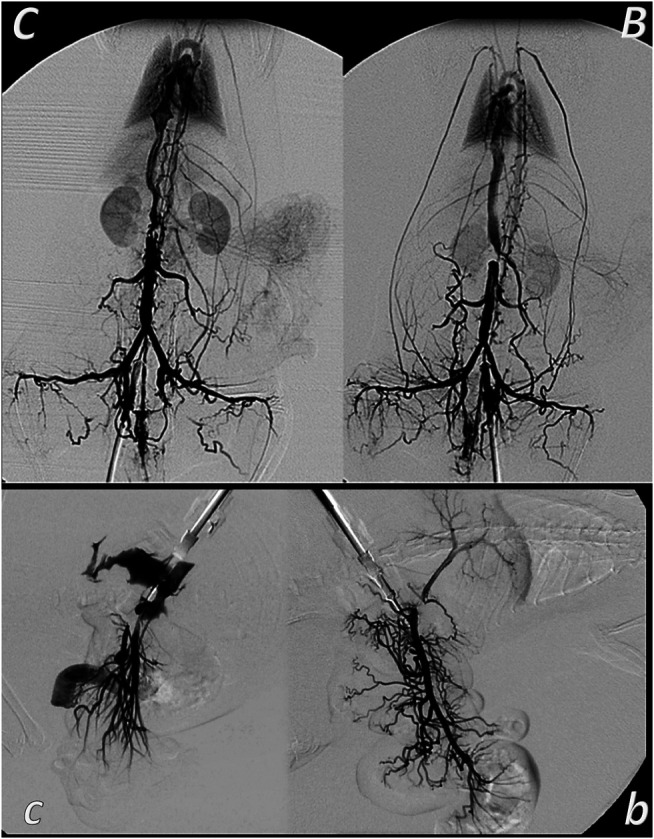
Venous occlusion and BPC 157 therapy. Given BPC 157 (as an abdominal bath) immediately before venography, at a particular point, venography demonstrated a rapid recruitment of the collaterals to bypass occlusion and reestablish blood flow. In the rats with infrarenally occluded inferior caval vein, venography in the inferior caval vein below the ligation shows that the left ovarian vein is rapidly presented as the major pathway. The other veins (such as epigastric veins, intercostal veins, mammary veins, iliolumbar veins, paraumbilical vein, azygos vein, and right ovarian vein) accordingly appear in BPC 157 rats (*B*), unlike in controls (*C*). Together, this means rapidly activated efficient compensatory pathways and the ligation-stop at the inferior caval vein efficiently bypassed (Vukojevic et al., 2018). Both kidneys and canal systems and confluence of the inferior caval vein to the right heart demonstrated that redistribution of otherwise trapped blood volume was rapidly achieved (Vukojevic et al., 2018). In the rats with occluded superior mesenteric vein, occlusion was made at the end of the superior mesenteric vein. Venography in superior mesenteric vein below the ligation shows that bypassing through inferior anterior pancreaticoduodenal vein and superior anterior pancreaticoduodenal vein to the pyloric vein toward the portal vein rapidly occurs in BPC 157 rats (*b*), unlike failed bypassing presentation in controls with occluded superior mesenteric vein (*c*).

The BPC 157 therapy of the bleeding disorders can be considered as an implementation and support of its wound healing effect (Seiwerth et al., 1997; Mikus et al., 2001; Sikiric et al., 2003; Xue et al., 2004a; Bilic et al., 2005; Seveljevic-Jaran et al., 2006; Tkalcevic et al., 2007; Huang et al., 2015) that can be purposefully extended (Stupnisek et al., 2012; Stupnisek et al., 2015). This particular balanced modulatory action in wound healing, which rapidly appears, along with this pentadecapeptide particular characteristics, may be even more interesting and more effective to be demonstrated in relation that in wounding, it decreases the bleeding. Namely, as curing of the wounds includes resolution of vessel constriction, the primary platelet plug, the secondary plug, and resolution of the clot (Stupnisek et al., 2012; Stupnisek et al., 2015) stabilize gastric pentadecapeptide BPC 157, which is effective in wound healing (Figures 1–6) (Seiwerth et al., 1997; Mikus et al., 2001; Sikiric et al., 2003; Xue et al., 2004a; Bilic et al., 2005; Seveljevic-Jaran et al., 2006; Tkalcevic et al., 2007; Huang et al., 2015); it also counteracts the bleeding disorders, amputation, organ perforation, and/or anti-coagulants application or major vessel occlusion (Stupnisek et al., 2012; Stupnisek et al., 2015; Drmic et al., 2018; Vukojevic et al., 2018; Gojkovic et al., 2020; Kolovrat et al., 2020). This reversal was seen also on the background of the disturbed prostaglandins- and NO-systems, prostaglandins synthesis inhibition as well as NO-overstimulation (NOS-substrate L-arginine) or NO-blockade (N(G)-nitro-L-arginine methyl ester (L-NAME)) (Stupnisek et al., 2012; Stupnisek et al., 2015). This should be taken along with its endothelium maintenance as the follow-up of its cytoprotection capability (Robert, 1979; Sikiric et al., 2010; Sikiric et al., 2018), along with the evidence that pentadecapeptide BPC 157 may prevent and/or attenuate or eliminate, thus, counteract both developing and already formed both arterial thrombosis (Hrelec et al., 2009; Gojkovic et al., 2020; Kolovrat et al., 2020), and venous thrombosis (Vukojevic et al., 2018; Gojkovic et al., 2020; Kolovrat et al., 2020). This therapy rapidly reversed the hind legs failure after abdominal aorta anastomosis (Hrelec et al., 2009). After occlusion of the inferior caval vein, accordingly, BPC 157 counteracts the whole Virchow triad (Vukojevic et al., 2018) and inferior caval vein syndrome (Vukojevic et al., 2018). At a particular point, venography demonstrated a rapid recruitment of the collaterals to bypass occlusion and reestablish blood flow (Vukojevic et al., 2018). In the rats with infrarenally occluded inferior caval vein, the left ovarian vein is rapidly presented as the major pathway (Figure 7). The other veins (such as epigastric veins, intercostal veins, mammary veins, iliolumbar veins, paraumbilical vein, azygos vein, and right ovarian vein) accordingly appear. Together, this means rapidly activated efficient compensatory pathways and the ligation stop at the inferior caval vein efficiently bypassed (Vukojevic et al., 2018). Both kidneys and canal systems and confluence of the inferior caval vein to the right heart demonstrated that the trapped blood volume is rapidly redistributed (Vukojevic et al., 2018). This commonly occurred with all of the used BPC 157 therapeutic regimens as well as at both early and advanced stages (Vukojevic et al., 2018). There is a similar beneficial effect in the rats with the Pringle maneuver, ischemia, reperfusion, and suprahepatic inferior caval vein occlusion (Budd–Chiari syndrome) (Gojkovic et al., 2020; Kolovrat et al., 2020). Thus, confronted with major vessels occlusion, BPC 157 commonly alleviates the peripheral vascular occlusion disturbances (Vukojevic et al., 2018; Gojkovic et al., 2020; Kolovrat et al., 2020) rapidly activating alternative bypassing pathways to adequately reestablish the blood flow (Duzel et al., 2017; Amic et al., 2018; Drmic et al., 2018; Vukojevic et al., 2018; Gojkovic et al., 2020; Kolovrat et al., 2020). Once the therapeutic effect begun, the therapeutic effect is continuous, and neither of the continuous ligation-induced disturbances reappeared (Vukojevic et al., 2018; Gojkovic et al., 2020; Kolovrat et al., 2020). Furthermore, direct vein injury, thrombosis, thrombocytopenia, and prolonged bleeding were all counteracted (Vukojevic et al., 2018; Gojkovic et al., 2020; Kolovrat et al., 2020). Trapped blood volume redistribution rapidly occurred throughout rapid presentation of collaterals (Vukojevic et al., 2018; Gojkovic et al., 2020; Kolovrat et al., 2020). Venous hypertension and arterial hypotension and tachycardia, rapidly presented, which were collectively attenuated or counteracted, emphasize BPC 157s therapeutic effects (Vukojevic et al., 2018; Gojkovic et al., 2020; Kolovrat et al., 2020). All these events mean that the counteraction of the full syndrome occurs (Vukojevic et al., 2018; Gojkovic et al., 2020; Kolovrat et al., 2020). This also means the counteracted oxidative stress was as a result of the lysis of endothelial cells (Vukojevic et al., 2018). Counteracted low NO-level in inferior caval vein and particular gene expression (i.e*. Egr, Nos, Srf, Vegfr, Akt1, Plcɣ,* and *Kras*) provide a likely special point to explain how the dysfunction and its counteraction is causal to, or result of (Vukojevic et al., 2018) (see *Genes Expression as Support*).

Finally, therapy as the recovering effect it has on occluded vessels (Duzel et al., 2017; Amic et al., 2018; Drmic et al., 2018; Vukojevic et al., 2018; Gojkovic et al., 2020; Kolovrat et al., 2020) bypassing the occlusion (Duzel et al., 2017; Amic et al., 2018; Drmic et al., 2018; Vukojevic et al., 2018; Gojkovic et al., 2020; Kolovrat et al., 2020) appears as the specific effect of BPC 157 in ischemia/reperfusion (Duzel et al., 2017; Amic et al., 2018; Drmic et al., 2018; Vukojevic et al., 2018; Gojkovic et al., 2020; Kolovrat et al., 2020). There is benefit in the inferior caval vein occlusion (Vukojevic et al., 2018; Gojkovic et al., 2020), also in colitis ischemia/reperfusion (Duzel et al., 2017), duodenal venous congestion lesions (Amic et al., 2018), and cecum perforation (Drmic et al., 2018), arising from BPC 157 therapy in addition to the deep vein thrombosis (Vukojevic et al., 2018; Gojkovic et al., 2020; Kolovrat et al., 2020). Recently, in the bile duct ligation-induced liver cirrhosis (Sever et al., 2019), no portal hypertension development and reversal of the already preexisting portal hypertension to normal values (Sever et al., 2019) have become possible.

Besides, BPC 157 had no effect on clotting parameters as shown in the previous amputation and/or anticoagulant studies (Stupnisek et al., 2012; Stupnisek et al., 2015; Vukojevic et al., 2018), while counteracting prolonged bleeding and thrombocytopenia (Stupnisek et al., 2012; Stupnisek et al., 2015; Vukojevic et al., 2018). Consistently, it was shown that BPC 157 maintains thrombocytes’ function (Konosic et al., 2019).

Here, the concluding evidence may be that BPC 157 counteracted adhesion formation after abdominal wall injury. Additionally, its counteracting effect occurred even with pre-existing adhesions (Berkopic Cesar et al., 2020).

Thus, with the damage of the peritoneum, two damaged peritoneal surfaces come into contact with each other, and the coagulation cascade is activated (Fortin et al., 2015). Contrarily, BPC 157 counteracts the whole Virchow triad (Vukojevic et al., 2018; Gojkovic et al., 2020; Kolovrat et al., 2020), venous thrombosis (Vukojevic et al., 2018; Gojkovic et al., 2020; Kolovrat et al., 2020), and arterial thrombosis (Hrelec et al., 2009; Gojkovic et al., 2020; Kolovrat et al., 2020). Also, BPC 157 administration attenuates prolonged bleeding and thrombocytopenia after amputation, organ perforation, and anticoagulant use or prolonged major vessel occlusion (Stupnisek et al., 2012; Stupnisek et al., 2015; Drmic et al., 2018; Vukojevic et al., 2018). Consequently, it may be likely that BPC 157 may also interfere with and reverse the process that would result in fusion to form a connection, for example, adhesion (Berkopic Cesar et al., 2020). Therefore, less adhesion formation as well as reversing existing adhesions, the restoration of normal tissue structure and function, suggest that BPC 157 may act with the temporary role of fibrin in healing without adhesions that must be purposefully degraded by the fibrinolytic system (Collen, 1980; Davey and Maher, 2017).

### Gene Expression as Support

In general, as before (Vukojevic et al., 2018), our focus was on the early and very early periods providing the general understanding of the precise coordination of cellular events, the formation and modification of the vascular system, and molecular signaling by numerous molecules which are responsible for fast activation and modulation of these events (Vukojevic et al., 2018).

For this purpose, the findings obtained in the rats with infrarenal occlusion of the inferior caval vein may be noteworthy (Vukojevic et al., 2018). The investigation was done at two particular intervals, at 1 and 24 h. Assessed were the inferior caval vein (which was occluded), right ovarian vein (which appears as blind vessel due to the infrarenal occlusion), and left ovarian vein (which serves as a bypassing pathway) (Vukojevic et al., 2018). With BPC 157 administration, these beneficial effects were combined with particular specificities with the altered genes’ expression (*Egr, Nos, Srf, Vegr, Plcɣ*, and *Kras*) or no change (*Akt1* continuously remained unchanged) (Vukojevic et al., 2018).

BPC 157 administration raises several questions regarding its therapeutic role in the process occurring in rats with obstructed vessels (Sikiric et al., 2014; Duzel et al., 2017; Vukojevic et al., 2018; Gojkovic et al., 2020; Kolovrat et al., 2020) and with vessels that used to be obstructed (Duzel et al., 2017; Kolovrat et al., 2020; Vukojevic et al., 2020). Rapid endothelial restoration and activation of the major collaterals (in addition, there are abundant anastomoses between individual vessels on both surfaces of the large intestine (Kachlik, et al., 2010)) may reorganize blood flow (Duzel et al., 2017; Vukojevic et al., 2018; Gojkovic et al., 2020; Kolovrat et al., 2020). Additionally, the rapid therapeutic effect of BPC 157 is evidenced by rapid reversal of the negative chain of events (Vukojevic et al., 2018). This included circumvention of the complex downhill syndrome, as well as a sudden decrease of blood supply, decreased NO levels in the inferior caval vein tissue, an immediate heavy loss of endothelial cells from the vascular wall, a lower eNOS production ability (Berra-Romani et al., 2013), and finally, the elimination of oxidative stress due to endothelial cell lysis (Rangan and Bulkley, 1993; Schiller et al., 1993). BPC 157 has been able to restore endothelial integrity and reverse most oxidative stress-induced damage (Duzel et al., 2017; Amic et al., 2018; Drmic et al., 2018; Vukojevic et al., 2018; Sever et al., 2019). Moreover, it largely interacts with the NO-system (Sikiric et al., 2014), the endothelium being significant in that demonstrates increased plasma NO-values, but normalized 3,4-methylenedioxyamphetamine (MDA)-values, as well as adequate endothelial NO-synthase (eNOS) system function (Duzel et al., 2017; Amic et al., 2018; Drmic et al., 2018; Vukojevic et al., 2018; Sever et al., 2019) and blood supply. After consideration of the aforementioned BPC 157–induced changes, several applications of BPC 157 with respect to its therapeutic effects on rats with preexisting (Duzel et al., 2017; Kolovrat et al., 2020; Vukojevic et al., 2020) or existing vessel obstruction (Sikiric et al., 2014; Duzel et al., 2017; Vukojevic et al., 2018; Gojkovic et al., 2020; Kolovrat et al., 2020) can be foreseen.

More specifically, this may be observed with respect to certain genes, including *Egr, Nos, Srf, Vegr, Akt1, Plcɣ*, and *Kras* genes (Vukojevic et al., 2018), all of which are responsible for the synthesis of factors necessary for a diverse set of processes. The factors have proadhesive, proinflammatory, and prothromobic roles, all of which become pertinent after vascular injury (Schweighofer et al., 2007; Ansari et al., 2015; Hinterseher et al., 2015). In these, the described BPC 157 efficacy is considered as an important demonstration of the confirmation that BPC 157 could ensure a particular specificity within the *Egr, Nos, Srf, Vegfr, Akt1, Plcɣ*, and *Kras* pathways (Vukojevic et al., 2018). After administering BPC 157 to rats with a ligated inferior caval vein, the beneficial effects induced by BPC 157 were complemented with altered *Egr, Nos, Srf, Vegr*, and *Kras* gene expression*.* Specifically, this included increased (*Egr, Nos, Srf,* and *Kras*) or decreased (*Egr, Vegfr,* and *Plcɣ*) gene expression, while *Akt1* gene expression remained unchanged (Vukojevic et al., 2018). These changes in gene expression are dependent on the time interval during which the genes were analyzed, as well as the type of vessel investigated. For example, increased levels of the *Egr* gene were observed at 1 h, *Nos*, *Srf*, and *Kras* genes demonstrated increased expression at 1 and 24 h in all vessels, and *Egr* expression was increased at 24 h in both ovarian veins. Likewise, decreased gene expression of *Egr* was evident at 24 h in the inferior caval vein, *Vegfr* expression was decreased at 1 h and 24 h in all vessels, and *Plcɣ* gene expression was decreased at 24 h in the inferior caval vein. *Akt1* remained unchanged in the inferior caval vein, as well as in the right ovarian vein and left ovarian vein (Vukojevic et al., 2018). Consequently, the aforementioned data suggest that particular pathways may have both local and systemic relevance. Collectively, these pathways may be responsible for the novel balance (of gene expression) achieved and ultimately, maintained. Finally, we should emphasize the rapid presentation of all of these effects. Obviously, they occurred before the initiation of angiogenesis (Sikiric et al., 1993; Sikiric et al., 2006; Sikiric et al., 2010; Sikiric et al., 2011; Sikiric et al., 2012; Sikiric et al., 2013; Seiwerth et al., 2014; Sikiric et al., 2014; Sikiric et al., 2016; Sikiric et al., 2017; Kang et al., 2018; Seiwerth et al., 2018; Sikiric et al., 2018; Gwyer et al., 2019; Park et al., 2020; Sikiric et al., 2020a; Sikiric et al., 2020b). Thus, we can assume that pathways additional to those involved in angiogenesis (Vukojevic et al., 2018) are associated with the increased expression, internalization of VEGFR2, and the activation of the VEGFR2-Akt-eNOS signaling pathway (Hsieh et al., 2017).

**FIGURE 8:**
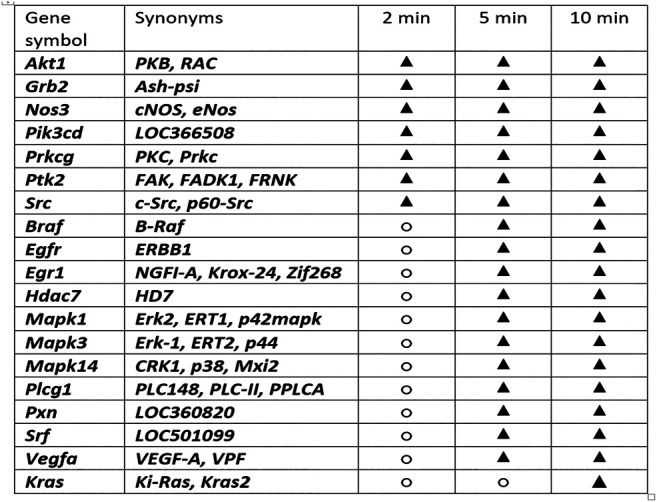
Gene expression analysis and BPC 157 therapy. With the therapy done immediately after wounding, we performed the *Akt1, Braf, Egfr, Egr1, Grb2, Hdac7, Kras, Mapk1, Mapk3, Mapk14, Nos3, Pik3cd, Plcg1, Prkcg, Ptk2, Pxn, Src, Srf*, and *Vegfa* genes expression analysis (○, no significant change in gene expression; ▲, increased gene expression) in the rats’ excision wound and in the skin and subcutaneous tissue, done at 2, 5, and 10 min following BPC 157 application. Thus, considering the quite rapid presentation of the BPC 157 beneficial effect in the wound healing, it is likely indicative that the expression of all of the genes *Akt1, Braf, Egfr, Egr1, Grb2, Hdac7, Kras, Mapk1, Mapk3, Mapk14, Nos3, Pik3cd, Plcg1, Prkcg, Ptk2, Pxn, Src, Srf,* and *Vegfa* is increased at the 10-min interval. An additional indicative point is sequential involvement. The increased expression of the *Akt1, Grb2, Nos3, Pik3cd, Prkcg, Ptk2,* and *Src* appears immediately at 2-min interval. *Braf, Egfr, Egr1, Hdac7, Mapk1, Mapk3, Mapk14, Plcg1, Prkcg, Ptk2, Pxn, Src, Srf,* and *Vegfa* increased expression is noted at 5 min. The increased expression of the *Kras* appears at 10 min. Of note, an enormous number of the interactions between the genes involved prevents a more definitive understanding of function insight outcome. However, the evidence is obtained that BPC 157 action initially affects expression of particular genes (i.e., *Akt1, Grb2, Nos3, Pik3cd, Prkcg, Ptk2, Src*). Then, it involves other set of the genes (*Braf, Egfr, Egr1, Hdac7, Mapk1, Mapk3, Mapk14, Plcg1, Prkcg, Ptk2, Pxn, Src, Srf,* and *Vegfa*). Subsequently, it concludes with additional genes set (i.e., *Kras*). Together, these may likely represent the complex way how the action, which will eventually resolve the lesion, may start and progress.

Furthermore, we additionally challenged the most immediate period after injury induction (skin defect) (i.e., 2, 5, and 10 min) (Figure 8) due to the evidence that BPC 157 did ensure a particular specificity within the pathways (at least, *Egr, Nos, Srf, Vegr, Akt1, Plcɣ*, and *Kras* genes) (Vukojevic et al., 2018). We emphasize the immediate relationship of the pentadecapeptide BPC 157 with the genes involved in cellular signaling pathways important for the regulation of angiogenesis. That point is supported with the extensive gene expression analysis. With the therapy done immediately after wounding, we performed the *Akt1, Braf, Egfr, Egr1, Grb2, Hdac7, Kras, Mapk1, Mapk3, Mapk14, Nos3, Pik3cd, Plcg1, Prkcg, Ptk2, Pxn, Src, Srf*, and *Vegfa* genes expression analysis in the rats’ excision wounds, in the skin, and subcutaneous tissue, done at 2, 5, and 10 min following BPC 157 application (Figure 8). Thus, considering the quite rapid presentation of the beneficial effect of BPC 157 in wound healing, it is likely indicative that the expression of all of the genes *Akt1, Braf, Egfr, Egr1, Grb2, Hdac7, Kras, Mapk1, Mapk3, Mapk14, Nos3, Pik3cd, Plcg1, Prkcg, Ptk2, Pxn, Src, Srf,* and *Vegfa* is increased at the 10-min interval. An additional indicative point is sequential involvement. The increased expression of the *Akt1, Grb2, Nos3, Pik3cd, Prkcg, Ptk2,* and *Src* appears immediately at 2-min interval. Increased expression of *Braf, Egfr, Egr1, Hdac7, Mapk1, Mapk3, Mapk14, Plcg1, Prkcg, Ptk2, Pxn, Src, Srf,* and *Vegfa* is noted at 5 min. The increased expression of the *Kras* appears at 10 min. Of note, an enormous number of the interactions between the genes involved obscure the final outcome. However, the evidence is obtained that BPC 157 action initially affects expression of particular genes (i.e., *Akt1, Grb2, Nos3, Pik3cd, Prkcg, Ptk2,* and *Src*). Then, it involves other set of the genes (*Braf, Egfr, Egr1, Hdac7, Mapk1, Mapk3, Mapk14, Plcg1, Prkcg, Ptk2, Pxn, Src, Srf,* and *Vegfa*). Subsequently, it concludes with additional set of genes (i.e., *Kras*). Together, these may likely represent the complex way of how the action, which will eventually resolve the lesion, may start and progress.

### The Effect on Other Tissues Healing as Support

**FIGURE 9:**
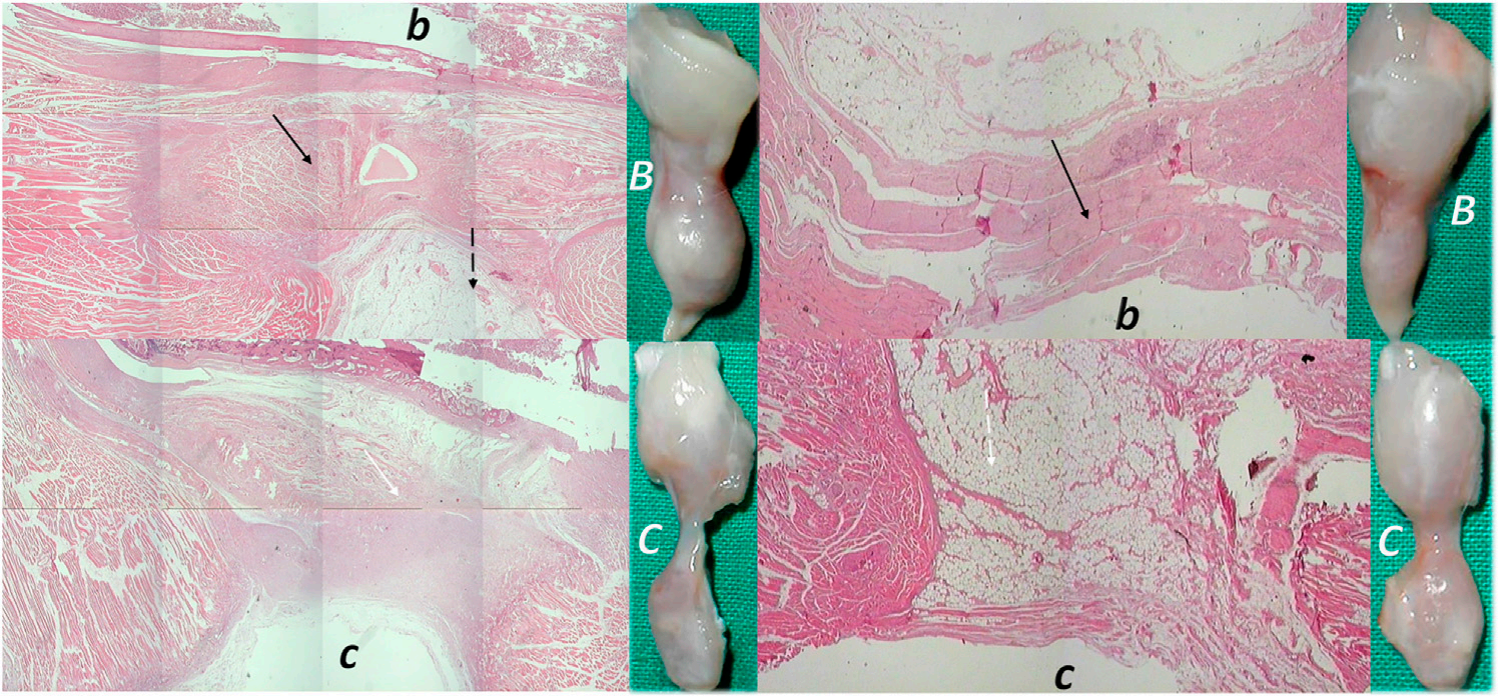
**(Left**, 14 days) **(right**, 28 days). Transected muscle and BPC 157 therapy (Staresinic et al., 2006). Quadriceps muscle was completely transected transversely 1.0 cm proximal to the patella means a definitive defect that cannot be compensated in rat. BPC 157 (10 μg, 10 ng, and 10 pg/kg) is given intraperitoneally, once daily; the first application 30 min posttransection, the final 24 h before sacrifice. Throughout the whole 72-day period, BPC 157 consistently improves all muscle-healing parameters (biomechanic, function, macro/microscopy/immunochemistry, finally presentation close to normal non-injured muscle, and no postsurgery leg contracture). Controls exhibit stumps grossly weakly connected (*C*), at postsurgery day 14 and 28, microscopically (HE, x10), at postsurgery day 14, gap filled with fibrous tissue (white arrow) (*c*), and at postsurgery day 28, a gap filled with fat tissue (dashed white arrow); incorporating few collagen strands is interposed between the transection stumps and unsuccessful attempts of muscle fibers to cross the gap can be observed (*c*). Contrarily, BPC 157 rats exhibit stumps grossly well connected, approaching to presentation of the normal non-injured muscle (*B*), microscopically, at postsurgery day 14 and at postsurgery day 28, broad muscle (black arrow) fibers connecting the stumps, while the fat tissue is much less present (*b*) (dashed black arrow).

**FIGURE 10:**
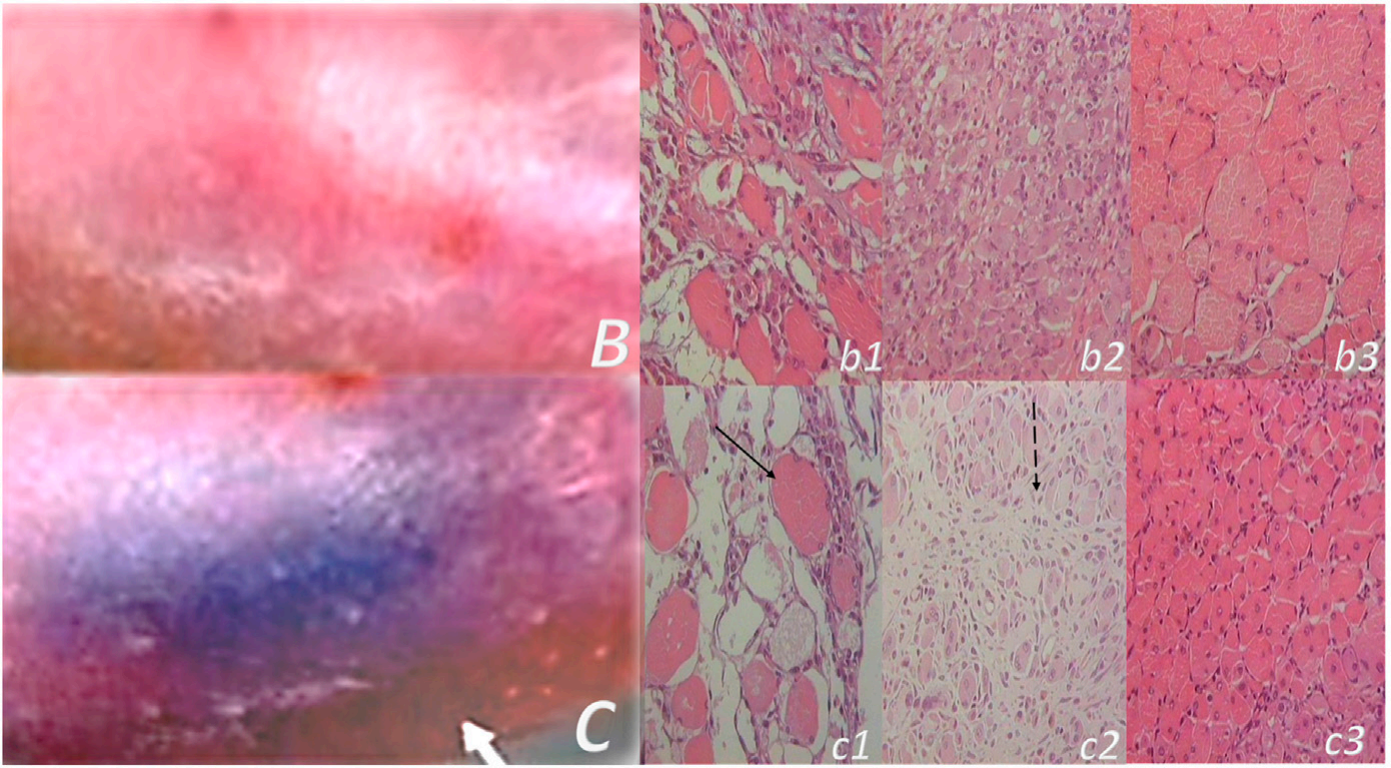
Muscle crush injury and BPC 157 therapy (Novinscak et al., 2008; Pevec et al., 2010). Force delivered was of 0.727 Ns/cm^2^ (impulse force 0.4653 Ns, kinetic energy 0.7217 J), to a maximum diameter of gastrocnemius muscle complex (GMC), about 2 cm proximal to the insertion of the Achilles tendon. Regimens with similar effectiveness included (i) BPC 157 dissolved in saline (10 μg, 10 ng/kg body weight), (ii) pentadecapeptide BPC 157 in neutral cream (1.0 or 0.01 μg dissolved in distilled water/g commercial neutral cream). Controls received saline (5.0 ml/kg) applied intraperitoneally and or commercial neutral cream applied as a thin cream layer at the site of injury. All animals were treated only once, immediately after injury, if killed and assessed after 2 h. Alternatively, the animals were treated once daily, receiving a final dose 24 h before death and/or assessment (walking, muscle function, and a macroscopic analysis) at days 1, 2, 4, 7, and 14. Gross posttraumatic hematoma presentation at 2 h after injury induction (*C*-control (white arrow), *B*-BPC 157 rats (thin cream layer at the site of the injury immediately after injury induction)). Microscopy assessment (*c1, b1* (postinjury day 4)*, c2, b2* (postinjury day 7), and *c3, b3* (postinjury day 14)). HE, x10. Controls. Severe atrophy with severe reduction of myocytes (black arrow) and no regeneration attempt, pronounced perimyocytic edema (postinjury day 4, *c1*); scarce to moderate regeneratory attempts in muscle with maturing granulation tissue (dashed black arrow) (postinjury day 7, *c2*); pronounced regeneration with a high number of smaller myocytes and some areas of scarring (postinjury day 14, *c3*). BPC 157. Clearly visible regenerative activity and less edema (postinjury day 4, *b1*); florid regenerative activity in myocytes with high number of relatively small myocytes and no scarring (postinjury day 7, *b2*); well-regenerated myocytes of appropriate size and very little scarring (postinjury day 14, *b3*).

**FIGURE 11:**
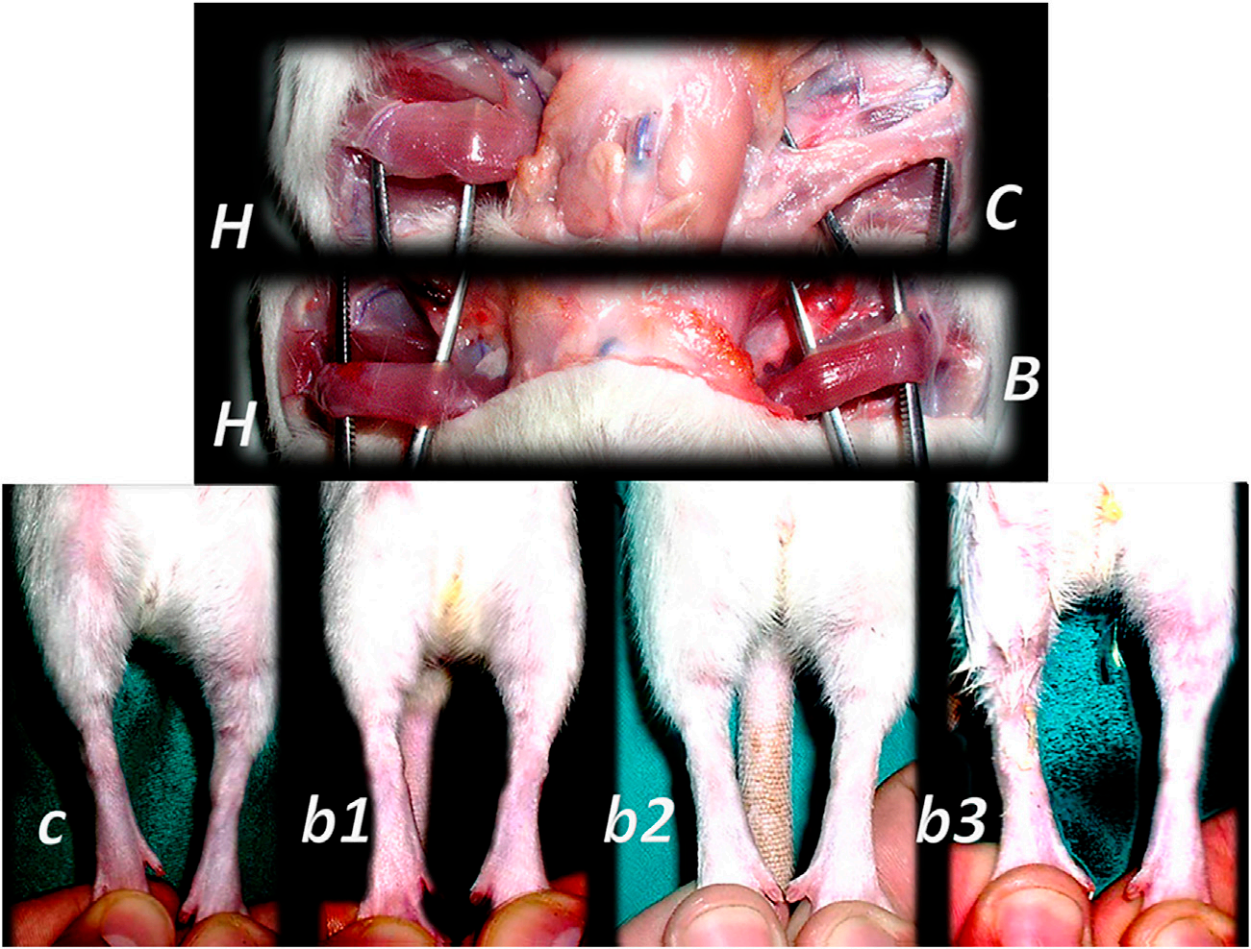
Denervated gracilis muscle and BPC 157 therapy **(upper)** (Mihovil et al., 2009). Transected muscle induced injured leg function failure as induced leg contracture and BPC 157 therapy **(lower)** (Staresinic et al., 2006). Presentation of the denervated gracilis muscle (*B, C*) and normal healthy gracilis muscle (*H*) in rats at 1 year after denervation. Characteristic denervated muscle presentation in controls (*C*). Counteraction by BPC 157 (10 μg/kg) therapy given per-orally, in drinking water (0.16 μg/ml, 12 ml/rat/day) till the sacrifice (*B*). Quadriceps muscle was completely transected transversely 1.0 cm proximal to patella to present a definitive defect that cannot be compensated in rats with a considerable injured leg contracture as presented at postsurgery day 21 in controls with maximal leg extension (*c*). Counteraction by various regimens of the BPC 157 (10 μg, 10 ng) therapy. Given intraperitoneally, once daily; the first application 30 min posttransection, the final 24 h before sacrifice (*b1*); per-orally, in drinking water (0.16 μg/ml, 0.16 ng/ml, and 12 ml/rat/day) till the sacrifice (*b2*); locally, thin layer of neutral cream 1 µg/1 g neutral cream once daily; the first application 30 min post-transection, the final 24 h before sacrifice (*b3*).

**FIGURE 12:**
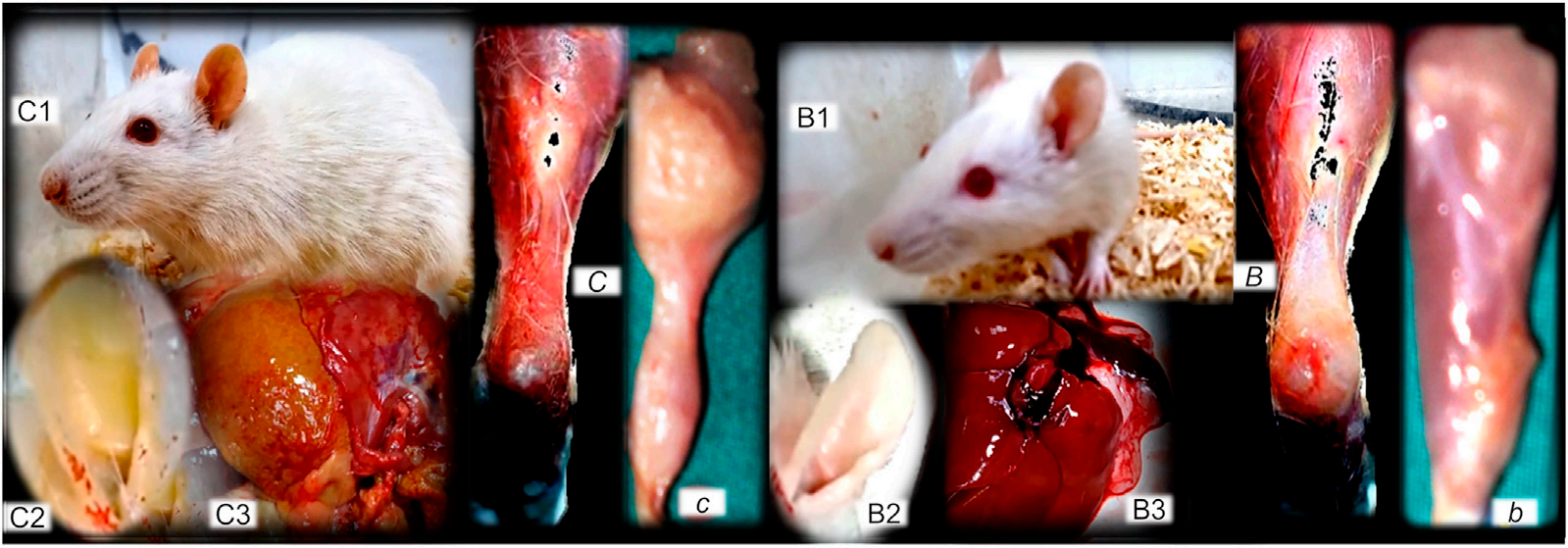
**(Left**, control), **(right**, BPC 157). Fibrosis and BPC 157 therapy. Summarized gross evidence. Presentation of the bile duct ligation rats (“yellow rats”) following 4 months of occlusion (as a model of the wound-healing response to chronic liver injury), gross rat presentation (C1), and after sacrifice, yellow ears (C2), and liver presentation (C3) (Sever et al., 2019). Possible analogy goes with the dermal, muscle (proximal and distal stump of quadriceps muscle poorly connected at day 72 after transection (*c*)), tendon (gap after tendon detached from calcaneus (*C*)) and ligament fibrosis and scar formation, and failed function (Mikus et al., 2001; Krivic et al., 2006; Staresinic et al., 2006; Cerovecki et al., 2010). In rats with bile duct ligation, BPC 157 counteracts cirrhosis and portal hypertension (gross rat presentation (B1), and after sacrifice, normal ears (B2), and liver presentation (B3)) much like it attenuates dermal, muscle (well-formed quadriceps muscle at day 72 after transection (*b*), tendon (tendon reattached to the calcaneous after detachement (*B*)), and ligament fibrosis and scar formation, and regained function (Mikus et al., 2001; Krivic et al., 2006; Staresinic et al., 2006; Cerovecki et al., 2010).

**FIGURE 13:**
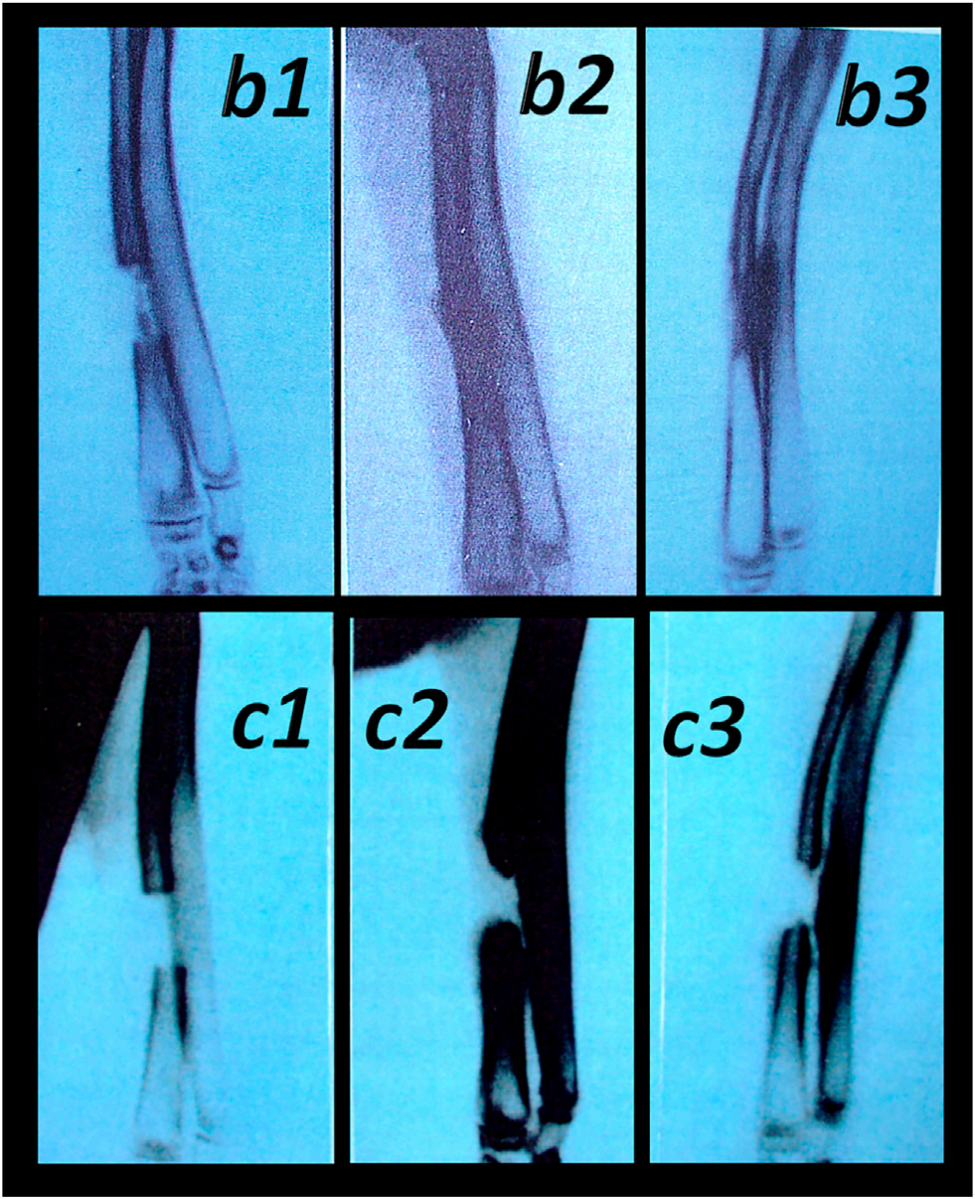
Healing of the segmental osteoperiosteal bone defect (0.8 cm, in the middle of the left radius) in rabbits (Sebecic et al., 1999). Incompletely healed defect in all controls (assessed during 6 weeks, in 2-weeks intervals (*c1* (week 2), *c2* (week 4), and *c3* (week 6)). Pentadecapeptide BPC 157 beneficial effect was consistently obtained (either percutaneously given locally (10 μg/kg) into the bone defect, or applied intramuscularly (intermittently, at postoperative days 7, 9, 14, and 16 at 10 μg/kg) or continuously (once per day, postoperative days 7–21 at 10 microg or 10 ng/kg)) (*b1* (week 2), *b2* (week 4), and *b3* (week 6)). Comparative regimens included percutaneous administration of autologous bone marrow locally (2 ml, postoperative day 7) as well as an autologous cortical graft inserted in the bone defect immediately after its formation. Saline-treated (2 ml intramuscularly (i.m.) and 2 ml locally into the bone defect), injured animals were used as controls (Sebecic et al., 1999).

Defining of the adequate skin wound healing effect (Seiwerth et al., 1997; Mikus et al., 2001; Sikiric et al., 2003; Xue et al., 2004a; Bilic et al., 2005; Seveljevic-Jaran et al., 2006; Tkalcevic et al., 2007; Huang et al., 2015) indicates its essential application in the other tissues healing, particularly, the muscle (Figures 9–12), tendon (Figure 12), ligament, and bone (Figure 13) (Sikiric et al., 1993; Sikiric et al., 2006; Sikiric et al., 2010; Sikiric et al., 2011; Sikiric et al., 2012; Sikiric et al., 2013; Seiwerth et al., 2014; Sikiric et al., 2014; Sikiric et al., 2016; Sikiric et al., 2017; Kang et al., 2018; Seiwerth et al., 2018; Sikiric et al., 2018; Gwyer et al., 2019; Park et al., 2020; Sikiric et al., 2020a; Sikiric et al., 2020b). Especially, BPC 157 exhibits both special muscle healing (i.e., after transection of major muscle (Figures 9, 11, 12), crush (Figure 10), and denervation (Figure 11) (Staresinic et al., 2006; Novinscak et al., 2008; Mihovil et al., 2009; Pevec et al., 2010)), and tendon healing (Staresinic et al., 2003; Krivic et al., 2006; Krivic et al., 2008) (i.e., after the Achilles tendon transection and detachment from calcaneal bone), or ligament healing (Cerovecki et al., 2010) (i.e., medial collateral ligament transection). In addition, along with function recovery (Figure 11), BPC 157 counteracts muscle disability related to various noxious procedures, after abdominal aorta anastomosis (Hrelec et al., 2009), L2-L3 compression (Perovic et al., 2019), severe electrolytes disturbances (Baric et al., 2016; Medvidovic-Grubisic et al., 2017), application of the succinylcholine (Stambolija et al., 2016), neuroleptics (Jelovac et al., 1999; Belosic Halle et al., 2017), or neurotoxin (MPTP, cuprizone) (Sikiric et al., 1999c; Klicek et al., 2013). Also, BPC 157 counteracts tumor cachexia (Kang et al., 2018). There are counteracted muscle wasting, significantly corrected deranged muscle proliferation as well as myogenesis, counteracted increase of the proinflammatory cytokines such as IL-6 and TNF-α, looking at muscle metabolism relevant to cancer cachexia as well as the changes in the expression of FoxO3a, p-AKT, p-mTOR, and P-GSK-3β (Kang et al., 2018). To illustrate the success of the application and the way of application efficacy, it may be instructive to mention the noted effect in the recovery of the transected sciatic nerve injury (providing the efficacy of the once intraperitoneal, intragastric application much like the application into the tube with inserted nerve stumps) (Gjurasin et al., 2010). In support, recently BPC 157 increased the survival rate of cultured enteric neurons and the proliferation rate of cultured enteric glial cells (Wang et al., 2019).

In the aforementioned study by Hsieh and collaborators (Hsieh et al., 2017) that focused on the ischemic muscle model (achieved via removal of the femoral artery), the pro-angiogenic effects of BPC 157 are evident, given the increased expression, internalization of vascular endothelial growth factor receptor 2 (VEGFR2), and the activation of the VEGFR2-Akt-eNOS signaling pathway (Hsieh et al., 2017). BPC 157 promotes angiogenesis in the chick choriallantoic membrane (CAM) assay and tube formation assay (Hsieh et al., 2017). Additionally, it counteracts rat hind limb ischemia by accelerating blood flow recovery and vessel number (Hsieh et al., 2017). BPC 157 upregulates VEGFR2 expression in rats with hind limb ischemia and endothelial cell culture. BPC 157–induced VEGFR2 internalization is combined with VEGFR2-Akt-eNOS activation. Of note, that BPC 157 effect is particular (i.e., activated the phosphorylation of VEGFR2, Akt, and eNOS signaling pathway) and does not need other known ligands or shear stress (Hsieh et al., 2017).

Also, this tissue-specific effect is demonstrated in tendon tissue-specific healing, which occurs without side effects that are present with other peptides (Seiwerth et al., 2018). For instance, ossicle formation in Achilles tendon tissue appears with osteogenic protein 1 (OP-1) (Forslund and Aspenberg, 1998). Contrary to this, it does not induce ossicle formation in tendon or ligament tissue (Staresinic et al., 2003; Krivic et al., 2006; Krivic et al., 2008). Specifically, BPC 157 accelerates tendon- and (Staresinic et al., 2003; Krivic et al., 2006; Krivic et al., 2008) ligament-healing (Cerovecki et al., 2010) and has a strong osteogenic effect the in non-union model (Sebecic et al., 1999) (and in other models, such as is observed with rat femoral head osteonecrosis (Gamulin et al., 2013), or alveolar bone loss (Keremi et al., 2009). Of note, in the tendon studies (Chang et al., 2011; Chang et al., 2014), BPC 157–induced promotion of the *ex vivo* outgrowth of tendon fibroblasts from tendon explants, cell survival under stress, and the *in vitro* migration of tendon fibroblasts are the effects mediated by the activation of the focal adhesion kinase (FAK)–paxillin pathway (Chang et al., 2011; Chang et al., 2014). Janus kinase (JAK) 2, the downstream signal pathway of growth hormone receptor, was activated in a time-dependent fashion via stimulation of the BPC 157–treated tendon fibroblasts with a growth hormone (Chang et al., 2011; Chang et al., 2014). Thus, the BPC 157–induced increase of growth hormone receptor in tendon fibroblasts may potentiate the proliferative effect of growth hormone and contribute to tendon healing (Chang et al., 2011; Chang et al., 2014).

Similarly, as the next wound-healing focus with BPC 157 therapy (Sever et al., 2019) administered per-orally, continuously in drinking water, or intraperitoneally, alleviated rats showed bile duct ligation (BDL) and counteracted BDL-induced liver cirrhosis (Sever et al., 2019). BPC 157 normalized MDA and NO-levels in the liver of BDL rats (Sever et al., 2019) and wound-healing response to chronic liver injury that may be accordingly modified (Figure 12) (Albanis and Friedman, 2001; Aller et al., 2008; Wynn, 2008; Sever et al., 2019). There were cured jaundice, weight loss, liver enlargement, microscopy (i.e., piecemeal necrosis, focal lytic necrosis, apoptosis and focal inflammation, and disturbed cell proliferation (Ki-67-staining), cytoskeletal structure in the hepatic stellate cell (α-SMA staining), collagen presentation (Mallory staining), and biochemistry presentation (Sever et al., 2019), and counteracted the increased NOS3 expression, interleukin (IL)-6, tumor necrosis factor (TNF)-α, and IL-1β levels. There were both prophylactic and therapy effects. Developing portal hypertension as well as established portal hypertension were both rapidly counteracted in BDL rats (i.e., BPC 157 per-orally, in drinking water, since the end of week 4) (Sever et al., 2019). The balanced collagen synthesis and counteracted hepatic fibrosis (considered as a model of the wound-healing response to chronic liver injury), are along with BPC 157s particular interaction with several molecular pathways (Tkalcevic et al., 2007; Chang et al., 2011; Cesarec et al., 2013; Chang et al., 2014; Huang et al., 2015; Hsieh et al., 2017; Kang et al., 2018; Vukojevic et al., 2018; Sever et al., 2019; Park et al., 2020; Vukojevic et al., 2020). In addition, there is a likely analogy (see for review i.e., (Seiwerth et al., 2018)) with the attenuated fibrosis and scar formation in other tissues (i.e., the skin, muscle, tendon, and ligament) (Mikus et al., 2001; Sikiric et al., 2003; Staresinic et al., 2003; Krivic et al., 2006; Staresinic et al., 2006; Krivic et al., 2008; Cerovecki et al., 2010). Also, they regained the dermal, muscle, tendon, and ligament functions (Mikus et al., 2001; Sikiric et al., 2003; Staresinic et al., 2003; Krivic et al., 2006; Staresinic et al., 2006; Krivic et al., 2008; Cerovecki et al., 2010). These, as mentioned before, occur in the BPC 157s healing of the severe injuries, such as the skin burns (Mikus et al., 2001; Sikiric et al., 2003) or muscle (Staresinic et al., 2006) or tendon (Staresinic et al., 2003; Krivic et al., 2006; Krivic et al., 2008) or ligament (Cerovecki et al., 2010) transection.

In this issue, the already mentioned Tkalcevic and collaborators’ study may be illustrative (Seveljevic-Jaran et al., 2006; Tkalcevic et al., 2007). A clear demonstration was obtained that BPC 157 stimulated both the egr-1 gene (critical in proliferation, differentiation, and inflammation during cholestatic liver injury, cytokine and growth factor generation, and early extracellular matrix (collagen) formation) (Kim et al., 2006; Zhang et al., 2011) and its co-repressor gene nab2 (Seveljevic-Jaran et al., 2006; Tkalcevic et al., 2007). Consequently, as also indicated in our other liver studies (Ilic et al., 2009; Ilic et al., 2010; Ilic et al., 2011a; Ilic et al., 2011b), BPC 157 (with nab2) may be a particular feedback-controlling mechanism.

The final point of the wound-healing focus with BPC 157 therapy (Seiwerth et al., 1997; Mikus et al., 2001; Sikiric et al., 2003; Xue et al., 2004a; Bilic et al., 2005; Seveljevic-Jaran et al., 2006; Tkalcevic et al., 2007; Huang et al., 2015) belongs to the controlling role of the pentadecapeptide BPC 157, considering that all of the described healing effects should avoid possible harmful outrage.

Illustratively, BPC 157 may interfere with NO-system activities (Sikiric et al., 2014) and counteract harmful events, arising from either NO-blockade (L-NAME-application) or L-arginine (NOS-substrate-application) (Sikiric et al., 2014). As an indicative example, without its own effect on normal blood pressure, it counteracts L-NAME hypertension as well as L-arginine hypotension (Sikiric et al., 1997a). Also, BPC 157 counteracts chronic heart failure (Lovric Bencic et al., 2004) or venous occlusion (Vukojevic et al., 2018)–induced hypotension (Lovric Bencic et al., 2004; Vukojevic et al., 2018) as well as hyperkalemia-induced hypertension (Barisic et al., 2013). Accordingly, BPC 157 provides the counteraction of the opposite effects of L-NAME (prothrombotic) and L-arginine (antithrombotic) application (Stupnisek et al., 2015). Likewise, BPC 157 may interfere with prostaglandin system activities, that is, it strongly counteracts NSAIDs toxicity (Sikiric et al., 2013), much like it counteracts developing and already formed adjuvant arthritis in rats (Sikiric et al., 1997c). Also, it strongly counteracts corticosteroid-induced adverse effects (i.e., eventual healing failure (Klicek et al., 2008; Krivic et al., 2008; Skorjanec et al., 2009; Pevec et al., 2010) and worsening, and immunosuppression (Sikiric et al., 2003).

That controlling point is also recognized in the most recent reviews (Gwyer et al., 2019; Sikiric et al., 2018). As indicated, despite the tumor-promoting effects of many growth factors and peptides (Gwyer et al., 2019; Sikiric et al., 2018), BPC 157 has been shown to inhibit and counteract uncontrolled cell proliferation (Ki-67 overproduction counteracted) and increased expression of VEGF and subsequent signaling pathways (Radeljak et al., 2004; Sever et al., 2019), thus avoiding and directly counteracting VEGF tumorigenesis (Radeljak et al., 2004). Furthermore, as mentioned (Gwyer et al., 2019), BPC 157 inhibits the growth of several tumor lines and can counteract tumor cachexia (Kang et al., 2018), a point combined, as mentioned, with counteracted pro-inflammatory and pro-cachectic cytokines such as IL-6, TNF-α, cancer cachexia–related pathways expression (i.e., FoxO3a, p-AKT, p-mTOR, and P-GSK-3β) (Kang et al., 2018). Also, BPC 157 may counteract cyclophosphamide toxicity (Luetic et al., 2017; Sucic et al., 2019), and in particular, BPC 157 consistently decreased elevated MDA values, while MDA itself, owing to its high cytotoxicity and inhibitory action on protective enzymes, is suggested to act as a tumor promoter and a cocarcinogenic agent (Seven et al., 1999; Bakan et al., 2002).

**FIGURE 14:**
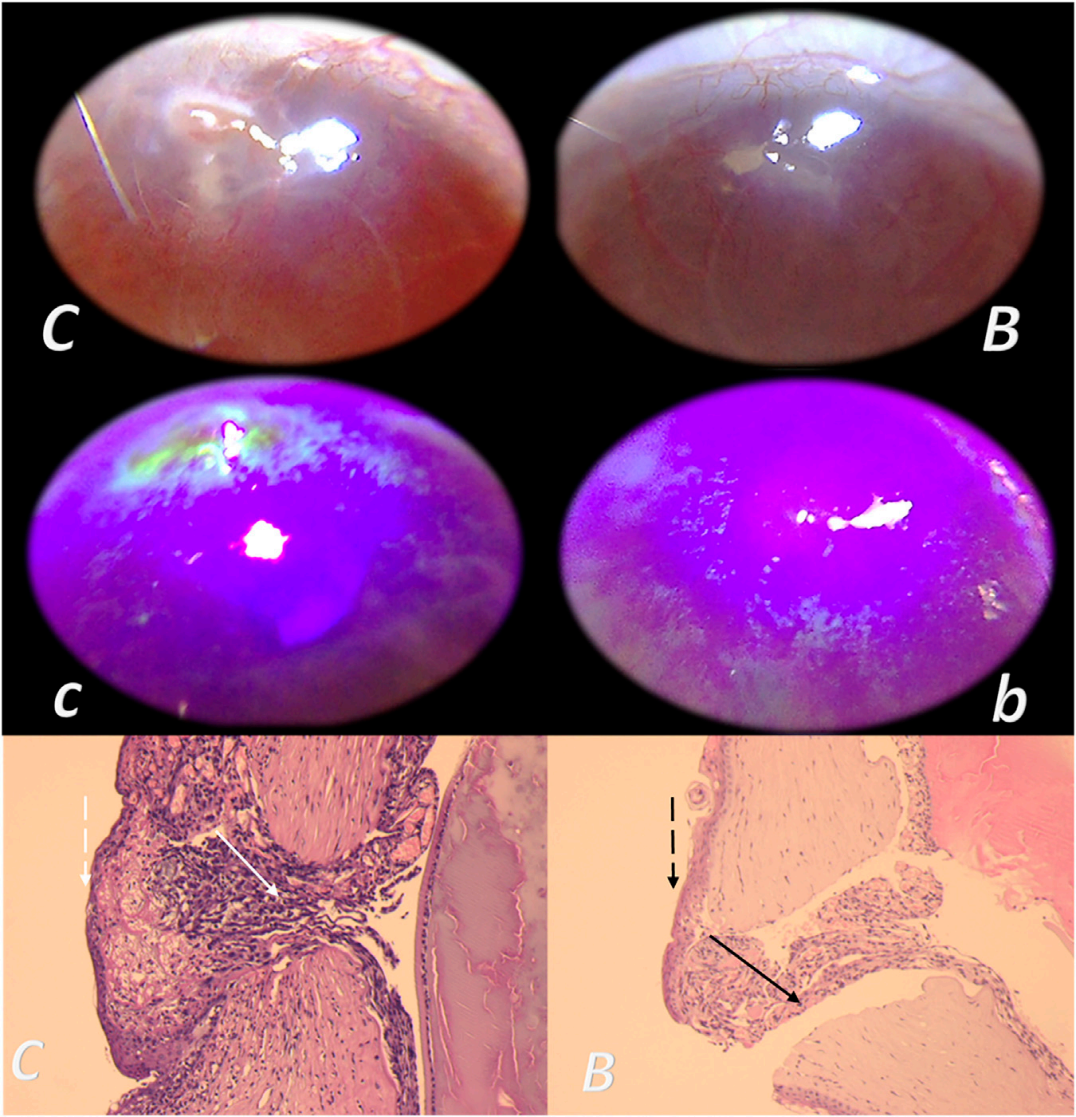
Corneal ulcer and BPC 157 therapy, ophathalmoscopy and microscopy presentation (Masnec et al., 2015). In deeply anesthetized rats, a penetrant linear 2-mm incision was made and two drops of 0.4% oxybuprocaine topical anesthetic were given (to inhibit possible eyelid reflex) in the paralimbal region of the left cornea at the 5 o’clock position with a 20-gauge MVR incision knife at 45° under an operating microscope. BPC 157 was dissolved in distilled water at 2 pg/ml, 2 ng/ml, and 2 μg/ml, and two eye drops were administered to the left eye in each rat immediately after induction of the injury and then every 8 h up to 120 h; controls received an equal volume of distilled water. Characteristic microscopy presentation (HE, x4) at 48 h after injury induction in control rats was much wider gap, not maturing and abundant granulation tissue, edematous with a lot of fibrin (clot-like) at the surface (white arrow). The surface epithelium is relatively unorganized and progressing over the gap showing a basal cell-like morphology (*C*) (dashed white arrow). In BPC 157–treated animals, the perforation channel is narrow, partly filled with well-vascularized but maturing, non-edematous granulation tissue (black arrow). The superficial epithelium progressing over the gap looks stratified (*B*) (dashed black arrow).

**FIGURE 15:**
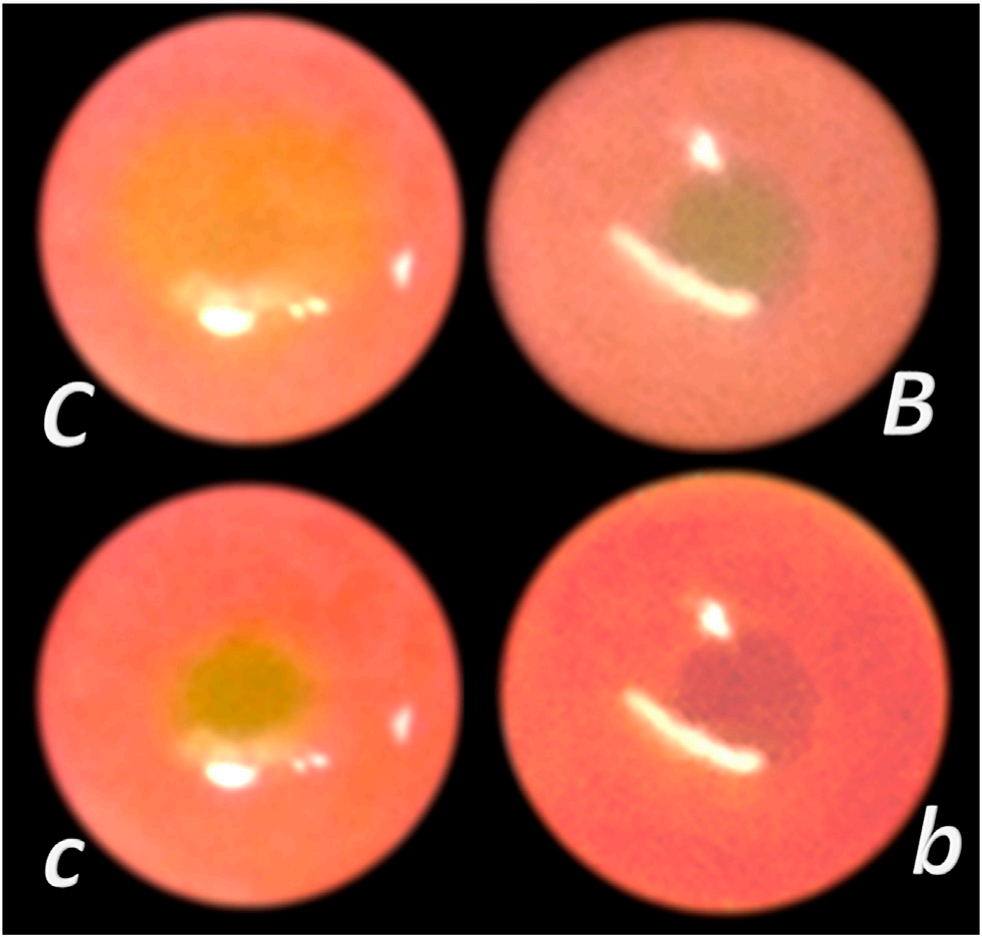
Total debridement of corneal epithelium and BPC 157 therapy (Lazic et al., 2005). Total debridement of corneal epithelium preformed in rats unilaterally and lesions stained (green) and photographed. Medication was distilled water (control group) or BPC 157 2 pg/ml, 2 ng/ml, and 2 μg/ml, two drops/rat eye started immediately after injury induction, every 8 h up to 40 h (i.e., at 0, 8, 16, 24, 32, and 40 h). Lesions presentation at 24 h in controls (*C*) and lesions attenuation in BPC 157 rats (*B*), lesions presentation at 32 h in controls (*C*) and lesions disappearance in BPC 157 rats (*B*).

Moreover, BPC 157 therapy generated from its distinctive healing effects in various tissues also functions as an eye drops therapy that heals perforating corneal ulcers in rats and rapidly regains corneal transparency (Figures 14, 15) (Masnec et al., 2015). Epithelial defects completely healed within three or four days (2 µg or 2 ng regimens, respectively), aqueous cells were not present after four or five days after injury. Regularly, in control rats, developed new vessels grew from the limbus to the penetrated area. Contrarily, generally no new vessels grew in BPC 157–treated rats (i.e., those that did form in the limbus did not make contact with the penetrated area) (Masnec et al., 2015), showing that BPC 157 therapy may acknowledge also “the angiogenic privilege” of the corneal avascularity as essential for corneal wound healing (Tshionyi et al., 2012). This may be the clue that to regain the tissue integrity after wounding, BPC 157 may accommodate the healing depending on the tissue and injury conditions.

## Summary

In conclusion, since the wound healing therapy with the standard angiogenic growth factors may be of essential importance, we presented an overview of the stable gastric pentadecapeptide BPC 157 and wound-healing issue.

Ultimately, this review challenges the general strategy that the skin wound healing (Seiwerth et al., 1997; Mikus et al., 2001; Sikiric et al., 2003; Xue et al., 2004a; Bilic et al., 2005; Seveljevic-Jaran et al., 2006; Tkalcevic et al., 2007; Huang et al., 2015), if properly accomplished, may be the principle largely generalized (see *Skin Wounds*). The success of such an undertaking depends, however, on a few particular, both practical and theoretical, considerations.

With the BPC 157 application (Sikiric et al., 1993; Sikiric et al., 2006; Sikiric et al., 2010; Sikiric et al., 2011; Sikiric et al., 2012; Sikiric et al., 2013; Seiwerth et al., 2014; Sikiric et al., 2014; Sikiric et al., 2016; Sikiric et al., 2017; Kang et al., 2018; Seiwerth et al., 2018; Sikiric et al., 2018; Gwyer et al., 2019; Park et al., 2020; Sikiric et al., 2020a; Sikiric et al., 2020b), this provides overcoming (Seiwerth et al., 2018) of the technical and practical limitations now presented as shortage in the corresponding standard angiogenic growth factors applicability and diverse delivery and carrier systems (see *Practical Application as Support*). In this, for the BPC 157 wound healing, we initially provided consistent argumentation for the accomplished successful skin wound healing (i.e., the combined triad collagen–inflammatory cells–angiogenesis was accordingly upgraded, appearing at earlier intervals, and the formation of granulation tissue containing mature collagen) (Seiwerth et al., 1997; Mikus et al., 2001; Sikiric et al., 2003; Xue et al., 2004; Bilic et al., 2005; Seveljevic-Jaran et al., 2006; Tkalcevic et al., 2007; Huang et al., 2015) (see *Skin Wounds*). With the consistent application of the peptide alone, this skin-healing evidence (Seiwerth et al., 1997; Mikus et al., 2001; Sikiric et al., 2003; Xue et al., 2004a; Bilic et al., 2005; Seveljevic-Jaran et al., 2006; Tkalcevic et al., 2007; Huang et al., 2015) was successfully applied to the other tissues healing (and thereby avoiding all shortages related to the peptide + carrier complex(es) (standard angiogenic growth factors application with diverse carriers)) (see *Practical Application as Support*). The arguments include the same dose range and same equipotent routes of application, regardless of the injury tested (Sikiric et al., 1993; Sikiric et al., 2006; Sikiric et al., 2010; Sikiric et al., 2011; Sikiric et al., 2012; Sikiric et al., 2013; Seiwerth et al., 2014; Sikiric et al., 2014; Sikiric et al., 2016; Sikiric et al., 2017; Kang et al., 2018; Seiwerth et al., 2018; Sikiric et al., 2018; Gwyer et al., 2019; Park et al., 2020; Sikiric et al., 2020a; Sikiric et al., 2020b), to induce a simultaneous healing, even in different tissues (and therefore the healing of diverse fistulas (Klicek et al., 2008; Skorjanec et al., 2009; Cesarec et al., 2013; Skorjanec et al., 2015; Baric et al., 2016; Grgic et al., 2016; Sikiric et al., 2020a)). This also can be applied to the healing of anastomoses, as evidenced by the simultaneous healing of both stumps of the anastomosis (i.e., various gastrointestinal anastomoses (Sikiric et al., 1993; Sikiric et al., 2006; Sikiric et al., 2010; Sikiric et al., 2011; Sikiric et al., 2012; Sikiric et al., 2013; Seiwerth et al., 2014; Sikiric et al., 2014; Sikiric et al., 2016; Sikiric et al., 2017; Kang et al., 2018; Seiwerth et al., 2018; Sikiric et al., 2018; Gwyer et al., 2019; Park et al., 2020; Sikiric et al., 2020a; Sikiric et al., 2020b), even those with large part of the bowel removal (Sever et al., 2009; Lojo et al., 2016), but also other tissue anastomoses, i.e., vessels (Numata et al., 2006) or nerve (Jelovac et al., 1999)) that occurs with the application of BPC 157. In addition, in BPC 157 short-bowel rats, the counteraction of the escalating short bowel syndrome provides that all three intestinal wall layers accordingly adapt in parallel with reaching the same weight gain as in normal rats (Sever et al., 2009) (see *Fistula Healing as Support*). The further proof of the concept includes much like the skin wound healing (Seiwerth et al., 1997; Mikus et al., 2001; Sikiric et al., 2003; Xue et al., 2004a; Bilic et al., 2005; Seveljevic-Jaran et al., 2006; Tkalcevic et al., 2007; Huang et al., 2015), the muscle, tendon, ligament, and bone healing (Sebecic et al., 1999; Staresinic et al., 2003; Krivic et al., 2006; Staresinic et al., 2006; Krivic et al., 2008; Novinscak et al., 2008; Keremi et al., 2009; Mihovil et al., 2009; Cerovecki et al., 2010; Pevec et al., 2010; Chang et al., 2011; Gamulin et al., 2013; Chang et al., 2014; Hsieh et al., 2017) (see *The Effect on Other Tissues Healing as Support*). The combining clue may be also the particular effect on blood vessels as the follow-up of its cytoprotection capability (Robert, 1979; Sikiric et al., 2010; Sikiric et al., 2018) (see *Therapy of Bleeding Disorders as Support*). The accomplishment of wound healing includes resolution of all four major events (vessel constriction, the primary platelet plug, the secondary plug, and resolution of the clot) (Stupnisek et al., 2012; Stupnisek et al., 2015). Therefore, stable gastric pentadecapeptide BPC 157 is effective in wound healing (Seiwerth et al., 1997; Mikus et al., 2001; Sikiric et al., 2003; Xue et al., 2004a; Bilic et al., 2005; Seveljevic-Jaran et al., 2006; Tkalcevic et al., 2007; Huang et al., 2015), much like it is effective also in counteracting bleeding disorders (Stupnisek et al., 2012; Stupnisek et al., 2015; Vukojevic et al., 2018), produced by amputation, and/or anti-coagulants application, or major vessel occlusion, along with the evidence that pentadecapeptide BPC 157 may prevent and/or attenuate or eliminate, thus, counteracting both arterial thrombosis (Hrelec et al., 2009; Gojkovic et al., 2020; Kolovrat et al., 2020) and venous thrombosis (Vukojevic et al., 2018; Gojkovic et al., 2020; Kolovrat et al., 2020). Consequently, the therapy signifies the recovering effect BPC 157 has on occluded vessels (Vukojevic et al., 2018; Gojkovic et al., 2020; Kolovrat et al., 2020; Vukojevic et al., 2020), bypassing the occlusion (Duzel et al., 2017; Amic et al., 2018; Drmic et al., 2018; Vukojevic et al., 2018; Gojkovic et al., 2020; Kolovrat et al., 2020; Vukojevic et al., 2020), appears as the specific effect of BPC 157 in ischemia/reperfusion (Duzel et al., 2017; Amic et al., 2018; Drmic et al., 2018; Vukojevic et al., 2018; Gojkovic et al., 2020; Kolovrat et al., 2020; Vukojevic et al., 2020). Finally, they reflect pathways likely additional to those involved in the angiogenesis (Vukojevic et al., 2018), recently associated in particular with the increased expression, internalization of VEGFR2, and the activation of the VEGFR2-Akt-eNOS signaling pathway (Hsieh et al., 2017) (see *Genes Expression as Support*), given the rapid presentation of all of these effects, which occurred before the process of angiogenesis could be initiated (Sikiric et al., 1993; Sikiric et al., 2006; Sikiric et al., 2010; Sikiric et al., 2011; Sikiric et al., 2012; Sikiric et al., 2013; Seiwerth et al., 2014; Sikiric et al., 2014; Sikiric et al., 2016; Sikiric et al., 2017; Kang et al., 2018; Seiwerth et al., 2018; Sikiric et al., 2018; Gwyer et al., 2019; Park et al., 2020; Sikiric et al., 2020a; Sikiric et al., 2020b). Thus, in rats with occluded blood vessels, at 1 and 24 h, BPC 157 demonstrated a particular specificity within the pathways (at least, *Egr, Nos, Srf, Vegfr, Akt1, Plcɣ*, and *Kras*) and vessels involved (Vukojevic et al., 2018). In the most immediate period after injury induction (skin defect) (i.e., 2, 5, and 10 min), BPC 157 increases *Akt1, Braf, Egfr, Egr1, Grb2, Hdac7, Kras, Mapk1, Mapk3, Mapk14, Nos3, Pik3cd, Plcg1, Prkcg, Ptk2, Pxn, Src, Srf,* and *Vegfa* gene expression in rats’ excision wounds in the skin. Finally, based on the noted beneficial effect, BPC 157 may balance collagen synthesis and affect hepatic fibrosis, as a model of the wound-healing response to chronic liver injury, which was modified (Albanis and Friedman, 2001; Aller et al., 2008; Wynn, 2008; Sever et al., 2019). Namely, the BPC 157 effect, in addition to the counteracted BDL-induced liver cirrhosis and portal hypertension (Sever et al., 2019) is also the attenuated dermal, muscle, tendon, and ligament fibrosis and scar formation, and regained function (Mikus et al., 2001; Sikiric et al., 2003; Staresinic et al., 2003; Staresinic et al., 2006; Krivic et al., 2008; Cerovecki et al., 2010) (see *The Effect on Other Tissues Healing as Support*). Controlling the role of BPC 157 likely involves its particular interaction with the major systems involved in wound healing, such as the NO-system (counteracted harmful events, arising from either NO-blockade or NOS-substrate application) (Sikiric et al., 2014) and the prostaglandins system (i.e., large extent of the counteracted NSAIDs toxicity (Sikiric et al., 2013) as well as counteracted developing and already formed adjuvant arthritis in rats (Sikiric et al., 1997c)). For corticosteroid-related systems, we should emphasize the counteracted eventual healing failure (Klicek et al., 2008; Krivic et al., 2008; Skorjanec et al., 2009; Pevec et al., 2010) and worsening, as well as immunosuppression (Sikiric et al., 2003). Finally, there is the evidence of BPC 157 generation of NO in *ex vivo* condition (Sikiric et al., 1997a; Turkovic et al., 2004), resistant to L-NAME, even in conditions when L-arginine does not work, which has some physiologic importance (Sikiric et al., 1997; Turkovic et al., 2004). Consequently, since it is formed constitutively in the gastric mucosa, stable, and present in human gastric juice (Sikiric et al., 1993; Sikiric et al., 2006; Sikiric et al., 2010; Sikiric et al., 2011; Sikiric et al., 2012; Sikiric et al., 2013; Seiwerth et al., 2014; Sikiric et al., 2014; Sikiric et al., 2016; Sikiric et al., 2017; Kang et al., 2018; Seiwerth et al., 2018; Sikiric et al., 2018; Park et al., 2020; Sikiric et al., 2020a; Sikiric et al., 2020b), along with suggested significance of NO-synthase and the basal formation of NO in stomach mucosa, greater than that seen in other tissues (Whittle et al., 1992), BPC 157 exhibits a general, effective competing with both L-arginine analogs (i.e., L-NAME) and L-arginine (Sikiric et al., 2014). Additionally, there is a controlling role of the pentadecapeptide BPC 157 against the tumor-promoting effects of many growth factors and peptides (Gwyer et al., 2019; Sikiric et al., 2018) as well as counteracting harmful escape from the initial healing effect (i.e., angiogenesis). VEGF tumorigenesis was avoided and counteracted (Sikiric et al., 1997c), and inhibition of the growth of several tumor lines and counteraction of tumor cachexia (Kang et al., 2018) and counteraction of the uncontrolled cell proliferation (Ki-67 overexpression counteracted) (Sever et al., 2019) were observed. BPC 157 stimulated both egr-1 and its co-repressor gene nab2 (Tkalcevic et al., 2007). Since egr-1 is critical in proliferation, differentiation, and inflammation during cholestatic liver injury, cytokine and growth factor generation and early extracellular matrix (collagen) formation (Kim et al., 2006; Zhang et al., 2011), BPC 157 (with nab2) may have an essential role as a particular feedback-controlling mechanism. In these terms, indicative is the rat corneal ulcer successfully closed with the BPC 157 eye drops and maintained corneal transparency (Figure 14) (Masnec et al., 2015). With BPC 157, corneal transparency was maintained and regained after total debridement of corneal epithelium (Figure 15) and dry eye syndrome in rats when BPC 157 counteracts the damaging effects of lacrimal gland extirpation (Lazic et al., 2005; Kralj et al., 2017).

Together, these findings indicate that with regard to wounds in operating instances, a BPC 157–defensive system exists that may accommodate the healing depending on the tissue and injury conditions. Of note, the possible contribution of the particular counteracting effect on the intravenous dextran- or egg white–induced anaphylactoid reaction (Duplancic et al., 2014) should also be considered, although not specifically mentioned in this review. Unlike histamine receptor antagonists (H1 and H2), BPC 157 has a strong beneficial effect in both rats and mice. Upper and lower lip and snout edema, as well as extreme cyanosis and edema of paws and scrotum were markedly attenuated. Poor respiration and fatalities were not observed (Duplancic et al., 2014). However, it remains to be seen when this particular balanced modulatory action will have further application in therapy.
